# All quiet on the western front? The evolutionary history of monogeneans (Dactylogyridae: *Cichlidogyrus, Onchobdella*) infecting a West and Central African tribe of cichlid fishes (Chromidotilapiini)[Fn FN1]

**DOI:** 10.1051/parasite/2023023

**Published:** 2023-07-04

**Authors:** Tanisha Moons, Nikol Kmentová, Antoine Pariselle, Tom Artois, Wim Bert, Maarten P.M. Vanhove, Armando J. Cruz-Laufer

**Affiliations:** 1 UHasselt – Hasselt University, Faculty of Sciences, Centre for Environmental Sciences, Research Group Zoology: Biodiversity and Toxicology, Agoralaan Gebouw D 3590 Diepenbeek Belgium; 2 Nematology Research Unit, Department of Biology, Ghent University K.L. Ledeganckstraat 35 9000 Ghent Belgium; 3 Department of Parasitology, Faculty of Science, University of South Bohemia České Budějovice 37005 Czech Republic; 4 ISEM, Université de Montpellier, CNRS, IRD 34095 Montpellier France; 5 Faculty of Sciences, Laboratory “Biodiversity, Ecology and Genome”, Research Centre “Plant and Microbial Biotechnology, Biodiversity and Environment”, Mohammed V University 10000 Rabat Morocco

**Keywords:** Machine learning, Sympatric speciation, Allopatric speciation, Host-parasite evolution, Maximum parsimony

## Abstract

Owing to the largely unexplored diversity of metazoan parasites, their speciation mechanisms and the circumstances under which such speciation occurs – in allopatry or sympatry – remain vastly understudied. Cichlids and their monogenean flatworm parasites have previously served as a study system for macroevolutionary processes, *e.g.*, for the role of East African host radiations on parasite communities. Here, we investigate the diversity and evolution of the poorly explored monogeneans infecting a West and Central African lineage of cichlid fishes: Chromidotilapiini, which is the most species-rich tribe of cichlids in this region. We screened gills of 149 host specimens (27 species) from natural history collections and measured systematically informative characters of the sclerotised attachment and reproductive organs of the parasites. Ten monogenean species (Dactylogyridae: *Cichlidogyrus* and *Onchobdella*) were found, eight of which are newly described and one redescribed herein. The phylogenetic positions of chromidotilapiines-infecting species of *Cichlidogyrus* were inferred through a parsimony analysis of the morphological characters. Furthermore, we employed machine learning algorithms to detect morphological features associated with the main lineages of *Cichlidogyrus*. Although the results of these experimental algorithms remain inconclusive, the parsimony analysis indicates that West and Central African lineages of *Cichlidogyrus* and *Onchobdella* are monophyletic, unlike the paraphyletic host lineages. Several instances of host sharing suggest occurrences of intra-host speciation (sympatry) and host switching (allopatry). Some morphological variation was recorded that may also indicate the presence of species complexes. We conclude that collection material can provide important insights on parasite evolution despite the lack of well-preserved DNA material.

## Abstract (fr)

En raison de la nature largement inexplorée de la diversité des parasites métazoaires, leurs mécanismes de spéciation et les circonstances dans lesquelles cette spéciation se produit—allopatrie ou sympatrie—restent très peu étudiés. Les cichlidés et leurs parasites Plathelminthes monogènes ont déjà servi de modèle pour l’étude des processus macro-évolutifs, par exemple pour le rôle des radiations d’hôtes de l’Afrique de l’Est sur les communautés de parasites. Ici, nous étudions la diversité et l’évolution des monogènes peu étudiées qui infestent une lignée de poissons cichlidés d’Afrique occidentale et centrale : les Chromidotilapiini, qui est la tribu de cichlidés la plus riche en espèces dans cette région. Nous avons examiné les branchies de 149 spécimens hôtes (27 espèces) provenant de musées d’histoire naturelle et mesuré systématiquement les caractères informatifs des pièces sclérifiées du hapteur et des organes copulateurs des parasites. Dix espèces de monogènes (Dactylogyridae : *Cichlidogyrus* et *Onchobdella*) ont été trouvées ; huit sont nouvelles pour la science et une est redécrite. Les positions phylogénétiques des espèces de *Cichlidogyrus* infectant les chromidotilapiines ont été déduites par une analyse de parcimonie des caractères morphologiques. En outre, nous avons utilisé des algorithmes d’apprentissage automatique pour détecter les caractéristiques morphologiques associées aux principales lignées de *Cichlidogyrus*. Bien que les résultats de ces algorithmes expérimentaux restent peu concluants, l’analyse de parcimonie indique que les lignées de *Cichlidogyrus* et d’*Onchobdella* de l’Afrique de l’Ouest et Central sont monophylétiques, contrairement aux lignées d’hôtes qui sont paraphylétiques. Plusieurs cas de partage d’hôtes suggèrent des occurrences de spéciation synxénique (sympatrie) et de changement d’hôte (allopatrie). Certaines variations morphologiques ont été enregistrées et peuvent également indiquer la présence de complexes d’espèces. Nous concluons donc que le matériel de collection peut fournir des informations importantes sur l’évolution des parasites malgré le manque d'ADN exploitable.

## Introduction

### Parasite speciation: sympatry or allopatry?

Parasitism is one of the most successful lifestyles in terms of extant species richness [89] and biomass [52]. Evolutionary processes in these organisms have recently received increased attention, especially in the context of emerging infectious diseases (see [8]). Yet most evolutionary processes in parasites remain poorly investigated. This limited knowledge stems from our limited understanding of the evolutionary history of most parasite taxa, with the majority of species remaining undescribed [39, 89, 91] or lacking DNA sequence data [91]. Despite these knowledge gaps, the lifestyle of parasites predicts several distinct characteristics different from most free-living animals, such as highly specialised feeding behaviour and shorter generation times [37]. Parasite populations are often larger in numbers but also more fragmented than populations of free-living organisms [20, 37]. These characteristics influence evolutionary rates and effective population size [37]. Parasites have a strong potential for speciation [13, 90, 94, 116], which arises from their narrow habitat selection that can be limited to single host species or even distinct microhabitats on a host species [25, 64], and the evolutionary arms races between hosts and parasites [45]. In some cases, these factors can lead to adaptive radiations (*i.e.*, explosive species formation), of which some of the most spectacular examples are displayed by parasites including anisakid nematodes [51] and endoparasitic snails that infect corals [24].

As with many other organisms, parasite speciation can occur in allopatry or sympatry [37, 64, 116]. The major criterion in distinguishing between allopatric and sympatric speciation is whether the barrier to gene flow is extrinsic or intrinsic [23]. Extrinsic barriers prevent the mating between parasites from different host species because of geography or vector specificity [7, 23]. When applying the concepts of the “island hypothesis” to parasites, host species and even individual hosts may be considered as the equivalent of separated habitat patches (“islands”) for free-living organisms. The physical and/or phylogenetic distance between host species and/or individuals determines the permeability of the barriers separating these habitats [64]. Parasite speciation can, therefore, be a direct consequence of host speciation (*co-speciation*) or occur as *accidental host switching* from one species to another (also referred to as *lateral transfer*), which represents a form of geographic isolation of small populations [37]. Sympatric speciation in parasites occurs in the absence of physical barriers but in the presence of intrinsic barriers, *e.g.*, different preferences for mating habitats and allelic incompatibilities within parasites infecting the same host species or individuals [70] (also referred to as *intra-host speciation*, *duplication*, or *synxenic speciation*). These barriers can result in the evolution of a sexual preference for individuals infecting the same host species [23, 64]. Sympatric speciation has also been suggested to result from particular ecological conditions that facilitate *host switching through active host selection* [34, 47] (not to be confused with *accidental host switching*, which is a passive mechanism, see above) such as the nutritional value of the host species, intensity of competition with other parasites, host defence mechanisms, and availability of microhabitats [9]. Host selection is an important factor in parasite speciation [37, 64]; for instance, the high motility of many plant-feeding insects allows these parasites to seek out preferential host species and individuals, while this choice is not available for many other less motile parasite groups that rely on passive transmissions and dispersal pathways. However, the role of host selection as a criterion for sympatric speciation remains a discussion point in the literature [37, 64] as the strict definition of sympatric speciation would potentially limit its applicability to only a few groups of metazoan parasites, such as plant-pathogenic insects [64]. The potentially high extinction rate of parasite species can further complicate inferring their evolutionary history [71, 87]. Consequently, distinguishing between allopatric and sympatric speciation in parasites can be challenging.

### Monogenean flatworms: a model of host-parasite evolution

Monogenean parasites have been suggested as a model system for studying the processes of parasite diversification because of their simple life cycle, morphological and ecological diversity [90], and the high species richness of some genera *e.g.*, *Dactylogyrus* Diesing, 1850 [1, 22], *Gyrodactylus* von Nordmann, 1832 [131, 132], and *Cichlidogyrus* Paperna, 1960 [12, 79]. Monogenean species are often restricted to a few closely related host species [90] or even to microhabitats on a single host species [25, 44, 63]. African cichlid fishes and the monogeneans belonging to *Cichlidogyrus* are one of most extensively studied fish-monogenean systems and have been proposed as a macroevolutionary model for host-parasite interactions [11, 86, 92, 125]. African cichlids are well-known for their spectacular adaptive radiations [109, 114, 118], and their role as a model for evolutionary research [114]. The cichlid-*Cichlidogyrus* species network is the most extensively described host-parasite network from a species-rich host radiation [10]. A recent meta-analysis counted 477 different host-parasite combinations in this study system [10].

Despite these extensive research efforts, many species of *Cichlidogyrus* remain undiscovered [11, 125]. Most cichlid species have not been examined for parasitic infections, although research spanning several decades has explored species of *Cichlidogyrus* from the East African cichlid radiations (*e.g.*, [26, 98, 99]) and from the economically relevant tilapias belonging to *Coptodon* Gervais, 1848 and *Oreochromis* Günther, 1889 [21, 40, 42, 77, 80, 92]. Extensive knowledge gaps remain, especially for species native to West and Central Africa.

### Chromidotilapiine cichlids: species-rich yet overlooked

Chromidotilapiini Greenwood, 1987 is the most species-rich tribe of cichlids of Central and West Africa. The tribe includes more species (62) than the tilapias belonging to Oreochromini Dunz & Schliewen, 2010 (59) and Coptodonini Dunz & Schliewen, 2013 (31) across Africa [19]. Nonetheless, the parasite diversity of the latter tribes has been far more extensively studied [11], mainly owing to the economic importance of some of their members [125]. Chromidotilapiines are riverine [111] and also one of the earliest diverging African cichlid lineages together with Tylochromini Poll, 1986, Pelmatochromini Greenwood, 1987, Hemichromini Hoedeman, 1947, and Heterochromidinae Kullander, 1998 [113]. These fishes are the only African cichlids that are not included in the haplotilapiines, a large monophyletic group containing all tilapia-like cichlids and all members of the East African radiations [16].

In terms of their evolutionary history, chromidotilapiines show strong allopatric patterns and their species divergences have likely been driven by ancient geographic processes rather than ecological specialisation [111]. Many species have somewhat restricted known geographical ranges, *e.g.*, species of *Teleogramma* Boulenger, 1899 as well as *Enigmatochromis lucanusi* Lamboj, 2009, and *Limbochromis robertsi* (Thys van den Audenaerde & Loiselle, 1971) [53, 111]*.* Chromidotilapiines are also often geographically separated from their congeners by the limits of river basins (*e.g.*, species of *Benitochromis* Lamboj, 2001*, Congochromis* Stiassny & Schliewen, 2007*, Nanochromis* Pellegrin, 1904*, Pelvicachromis* Thys van den Audenaerde, 1968 [54, 56, 120], and *Thysochromis emili* Walsh, Lamboj & Stiassny, 2020 [128]). Furthermore, representatives of different genera appear to occupy similar ecological niches in their respective geographical ranges (*e.g.*, the sand-dwellers *Parananochromis longirostris* (Boulenger, 1903) and species of *Nanochromis*; see [111] and references therein for more examples), which seems indicative of allopatric speciation. Phylogenetic analyses highlight that these similar niches in different geographical areas also produced morphologically similar species groups, which have been assigned to the same genera as a consequence. These groups include *Chromidotilapia* sensu stricto in Central Africa and the “*Chromidotilapia guntheri* group” in West Africa, and *Pelvicachromis* sensu stricto and species of *Pelvicachromis* from the Upper Guinea region [111]. Some species distributed over large areas have, in fact, been found to consist of groups of morphologically similar species separated by geographical barriers, *e.g.*, a population of *Pelvicachromis taeniatus* Boulenger, 1901 from Cameroon was found to be morphologically similar to, yet distinct from populations from Benin and Nigeria, therefore, the former was reassigned the name *P. kribensis* Boulenger, 1911 [56]*.*

Among the 78 described species belonging to Chromidotilapiini, parasites of only three species [*Chromidotilapia guntheri* (Sauvage, 1882), *Parananochromis caudifasciatus* (Boulenger, 1913) and *Benitochromis batesii* (Boulenger, 1901)] have been reported [2, 15, 72]. In the present study, we investigate the morphological evolution of the monogenean fauna of 27 species belonging to the genera *Chromidotilapia*, *Congochromis*, *Divandu*, *Nanochromis*, *Parananochromis*, *Pelvicachromis,* and *Thysochromis.* As these species have not previously been examined for parasites, we expect to find new species as monogeneans express a high level of host specificity [127]. We expect to detect strong allopatric speciation patterns similar to those observed in the host lineages. The present study will expand our knowledge on cichlid-*Cichlidogyrus* interactions and the evolutionary history of *Cichlidogyrus*, one of the most species-rich genera of parasites on the African continent.

## Material and methods

### Parasite collection and morphological examination

**Figure 1:**
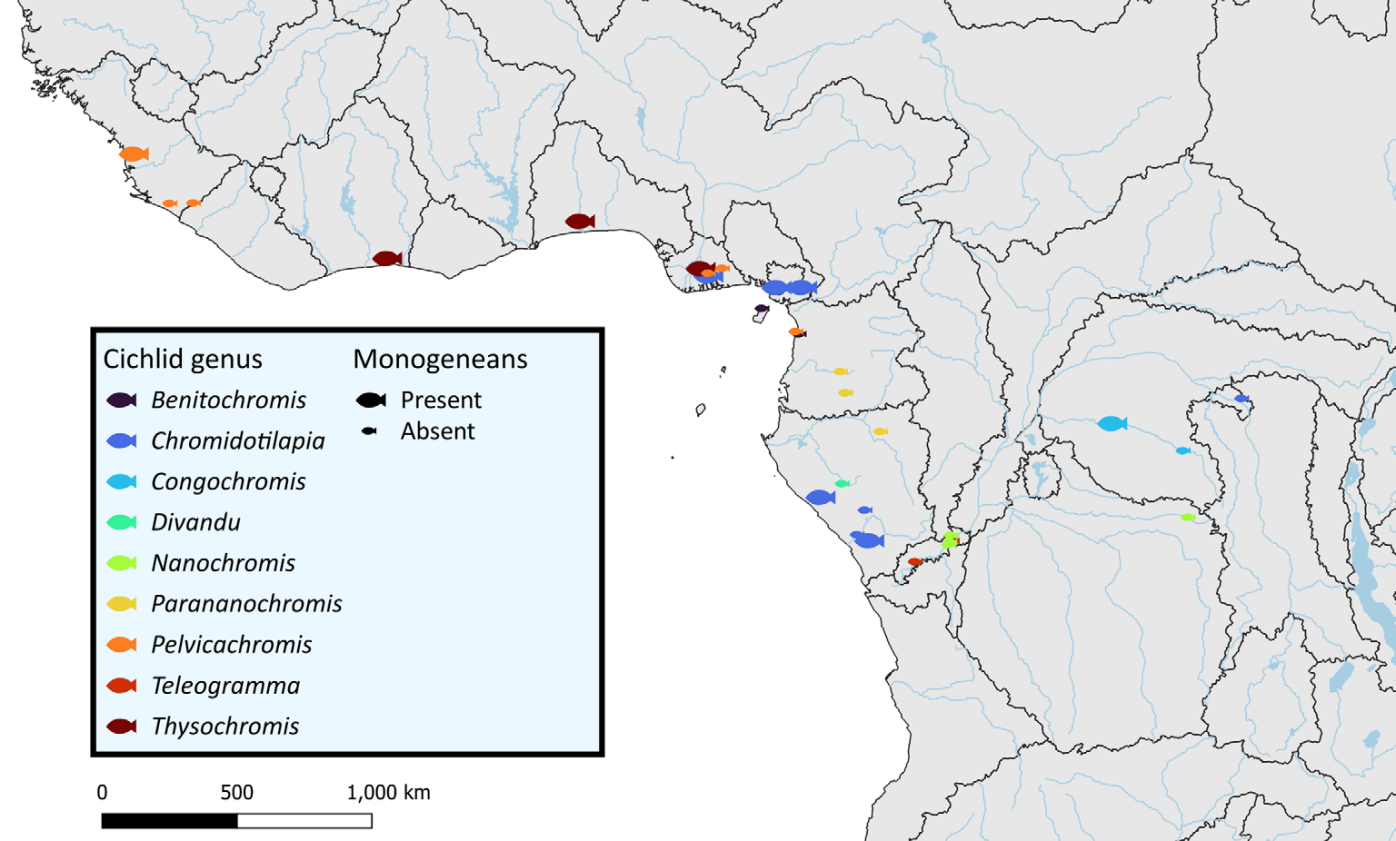
Sampling locations of chromidotilapiine cichlids across Central and West Africa with the presence of monogeneans indicated by size of the symbols. Borders indicate limits of freshwater ecoregions according to Thieme *et al.* [121].

Fish specimens were obtained from the ichthyological collection of the Royal Museum for Central Africa (RMCA) (Table 1). The gills of 149 individuals belonging to 27 species of Chromidotilapiini collected from several locations in West and Central Africa (Fig. 1) were dissected and subsequently stored in 100% ethanol. The gills were screened for the presence of monogenean infections under a stereomicroscope. Parasite specimens were mounted on slides with a drop of Hoyer’s medium [36] for morphological identification. Parasite identification and description were conducted using a Leica DM 2500 LED microscope (Leica Microsystems, Wetzlar, Germany) at 400× and 1000× magnification. High-resolution images were taken through the software LasX v3.6.0 (Leica Microsystems, Wetzlar, Germany). Type material was deposited in the invertebrate collection of the Royal Museum for Central Africa (Tervuren, Belgium) (RMCA_VERMES_44366–44602), the collection of the research group Zoology: Biodiversity and Toxicology of Hasselt University (Diepenbeek, Belgium) (HU 842–853), the Finnish Museum of Natural History (Helsinki, Finland) (MZH http://id.luomus.fi/KN.37258–http://id.luomus.fi/KN.37274), the Iziko South African Museum (Cape Town, South Africa) (SAMC-A095104–A095122), and the Musée National d’Histoire Naturelle (Paris, France) (MNHN HEL1906–1922).

**Table 1:** Sampling sites and dates of host specimens in the collection of the Royal Museum for Central Africa (RMCA) in Tervuren, Belgium, and monogenean gill parasites found on their gills.

Host	RMCA accession number	# samples	# infected	Collection date	Country	Locality	Latitude	Longitude	Parasite	# parasites
*Chromidotilpia elongata* Lamboj	RMCA_Vert_1991.068.P.2121-2125	1	1	30/07/1991	Republic of the Congo	Mavemba River, tributary of Loukoula on the right bank, 2 km downstream from Mpounga	−4.283	12.450	*Cichlidogyrus ophioglossus* n. sp.	4
									*Cichlidogyrus gnomon* n. sp.	2
*Chromidotilapia guntheri* (Sauvage)	RMCA_Vert_1973.005.P.4955-4978	4	3	24/10/1966	Cameroon	Lake Barombi-Kotto	4.467	9.250	*Cichlidogyrus dibangoi* n. sp.	1
									*Cichlidogyrus ataikputu* n. sp.	8
									*Cichlidogyrus tilapiae*	7
*Chromidotilapia guntheri* (Sauvage)	RMCA_Vert_1991.010.P.0542-0582	8	7	1–10/12/1990	Nigeria	New Calabar river, Akpor	4.867	6.900	*Cichlidogyrus ataikputu* n. sp.	9
									*Cichlidogyrus tilapiae*	2
									*Onchobdella krachii*	29
*Chromidotilapia kingsleyae* Boulenger	RMCA_Vert_2002.006.P.2722-2768	3	3	19/09/2001	Gabon	streamlet, affluent of Moukalaba River, Nyanga basin	−2.783	10.767	*Cichlidogyrus ophioglossus* n. sp.	133
*Chromidotilapia linkei* Staeck	RMCA_Vert_1992.144.P.0250-0261	1	1	07/11/1990	Cameroon	road Yabassi–Yingui	4.468	10.135	*Cichlidogyrus dibangoi* n. sp.	3
									*Cichlidogyrus ataikputu* n. sp.	8
									*Onchobdella krachii*	53
									*Cichlidogyrus tilapiae*	15
*Congochromis dimidiatus* (Pellegrin)	RMCA_Vert_P.174947-174968	4	1	29/09/1969	Democratic Republic of the Congo	Boende, Tshuapa Province	−0.233	20.833	*Cichlidogyrus tshuapa* n. sp.	2
*Pelvicachromis roloffi* (Thys van den Audenaerde)	RMCA_Vert_1973.010.P.6699-6703	2	1	05/04/1969	Sierra Leone	Kamaranka, near Rokupr 10–15 km, road Rokupr-Kambia	9.07	−12.93	*Cichlidogyrus* sp. ‘*Pelvicachromis roloffi*’	1
*Thysochromis ansorgii* (Boulenger)	RMCA_Vert_1984.022.P.0012-0014	1	1	03/1984	Nigeria	Oshika, 10 km North–West of Ahoada	5.117	6.633	*Onchobdella macrohamuli* n. sp.	2
									*Onchobdella yemojae* n. sp.	4
*Thysochromis ansorgii* (Boulenger)	RMCA_Vert_1973.005.P.4470-4476	2	2	13/09/1966	Côte d’Ivoire	Attingué, Agnébi Basin	5.470	−4.183	*Cichlidogyrus thysochromis* n. sp.	1
									*Onchobdella macrohamuli* n. sp.	2
									*Onchobdella yemojae* n. sp.	19
*Thysochromis ansorgii* (Boulenger)	RMCA_Vert_1973.005.P.4478-4503	5	3	14/10/1966	Benin	Whedda, River Ouémé	6.750	2.467	*Onchobdella macrohamuli* n. sp.	1
									*Onchobdella yemojae* n. sp.	5
*Divandu albimarginatus* Lamboj & Snoeks	RMCA_Vert_2001.070.P.2843-2867	4	0	21/02/2001	Gabon	streamlet 9km off Mitzic en route to Na	0.8175	11.62		
*Divandu albimarginatus* Lamboj & Snoeks	RMCA_Vert_1999.090.P.2083-2214	26	0	29/08/1998	Gabon	stream crossing road Bongolo- Mbélénaletembé, Ngounié-Ogooué basin	−2.320	11.501		
*Benitochromis batesii* (Boulenger)	RMCA_Vert_1992.144.P.0073-0109	7	0	26/12/1989	Cameroon	Bidou II, Meyo River, close to Kribi	2.850	10.017		
*Teleogramma brichardi* Poll	RMCA_Vert_P.177679-177684	2	0	1967	Democratic Republic of the Congo	Pool Malebo, Kinshasa	−4.300	15.300		
*Parananochromis caudifasciatus* (Boulenger)	RMCA_Vert_2001.070.P.2880-2903	4	0	25/02/2001	Gabon	Mintoumou, swamp close to the village Engone	1.550	11.440		
*Benitochromis finleyi* (Trewavas)	RMCA_Vert_1978.046.P.0135-0146	2	0	02/02/1968	Equatorial Guinea	Bioko Island, Fernando Po, Río Timbabe, stagnant pools in dry river	3.733	8.733		
*Parananochromis gabonicus* (Trewavas)	RMCA_Vert_2001.070.P.2907-2928	4	0	21/02/2001	Gabon	Streamlet 9km away from Mitzic on the way to Na	0.818	11.62		
*Teleogramma gracile* Boulenger	RMCA_Vert_1976.017.P.0024-0033	2	0	15/07/1973	Democratic Republic of the Congo	Congo River mainstream, near Bulu, West of Luozi	−5.017	14.017		
*Chromidotilapia kingsleyae* Boulenger	RMCA_Vert_1990.057.P.0881-0903	4	0	10/10/1990	Republic of the Congo	Loulimba River, village Doumanga III, road Bénai to Kakamoeka, 9km away from Kakamoeka	−4.083	12.017		
*Parananochromis longirostris* (Boulenger)	RMCA_Vert_2002.006.P.3133-3154	4	0	10/09/2001	Gabon	Loa Loa, Ivindo River, Ogôoué Basin	−0.521	12.823		
*Chromidotilapia mamonekenei* Lamboj	RMCA_Vert_2005.036.P.0432-0445	2	0	17/08/2006	Republic of the Congo	Mouhoula River at Loubetsi, Kouilou-Niari Basin	−3.237	12.287		
*Nanochromis nudiceps* (Boulenger)	RMCA_Vert_P.174305-174313	2	0	1–31/03/1959	Democratic Republic of the Congo	Lodja, Sankuru, Kasaï region	−3.483	23.433		
*Nanochromis nudiceps* (Boulenger)	RMCA_Vert_P.118107-118112	1	0	24/09/1957	Democratic Republic of the Congo	Pool Malebo, Kinsuka, rapids at the exit of the pool	−4.333	15.217		
*Nanochromis parilus* Roberts & Stewart	RMCA_Vert_P.118101-118106	1	0	17/08/1954	Democratic Republic of the Congo	Pool Malebo	−4.100	15.250		
*Nanochromis parilus* Roberts & Stewart	RMCA_Vert_P.98018-98026	3	0	12/08/1954	Democratic Republic of the Congo	Tsabuka, Congo rapids, Kinshasa	−4.433	15.167		
*Pelvicachromis pulcher* (Boulenger)	RMCA_Vert_1990.019.P.0463-0490	3	0	15/05/1989	Nigeria	3 km South of Isiokpo, New Calabar system	4.950	6.883		
*Pelvicachromis roloffi* (Thys van den Audenaerde)	RMCA_Vert_1973.010.P.6882-6885	1	0	10/05/1969	Liberia	Bombo junction, swamps and tributaries ±16km East of Mano, road Mano to Bomi Hills	7.367	−10.883		
*Pelvicachromis roloffi* (Thys van den Audenaerde)	RMCA_Vert_1973.010.P.6830-6847	3	0	16/04/1969	Sierra Leone	Pujehun, Waanje River and its tributaries marigots up- and downstream of the bridge at the level of the Gobaru hamlet	7.350	−11.700		
*Pelvicachromis sacrimontis* Paulo	RMCA_Vert_P.138748-138755	1	0	16/10/2007	Nigeria	Aba	5.117	7.367		
*Chromidotilapia schoutedeni* (Poll & Thys van den Audenaerde)	RMCA_Vert_1996.040.P.0001-0008	1	0	06/06/1995	Democratic Republic of the Congo	Ngene-Ngene River, road to Buta, km 16 in Kisangani	0.626	25.286		
*Congochromis squamiceps* (Boulenger)	RMCA_Vert_P.175561-175570	2	0	1955	Democratic Republic of the Congo	Equateur Region, Ikela, Tshuapa River	−1.183	23.267		
*Pelvicachromis subocellatus* (Günther)	RMCA_Vert_1999.055.P.1848-1858	2	0	08/10/1998	Gabon	Moukalaba River, 22km downstream from Douano (no coordinates found)				
*Pelvicachromis taeniatus* (Boulenger)	RMCA_Vert_1977.017.P.1264-1270	1	0	02/1973	Cameroon	Kribi, Kienke River	2.933	9.900		

### Morphometrics, missing data, and principal component analysis

**Figure 2:**
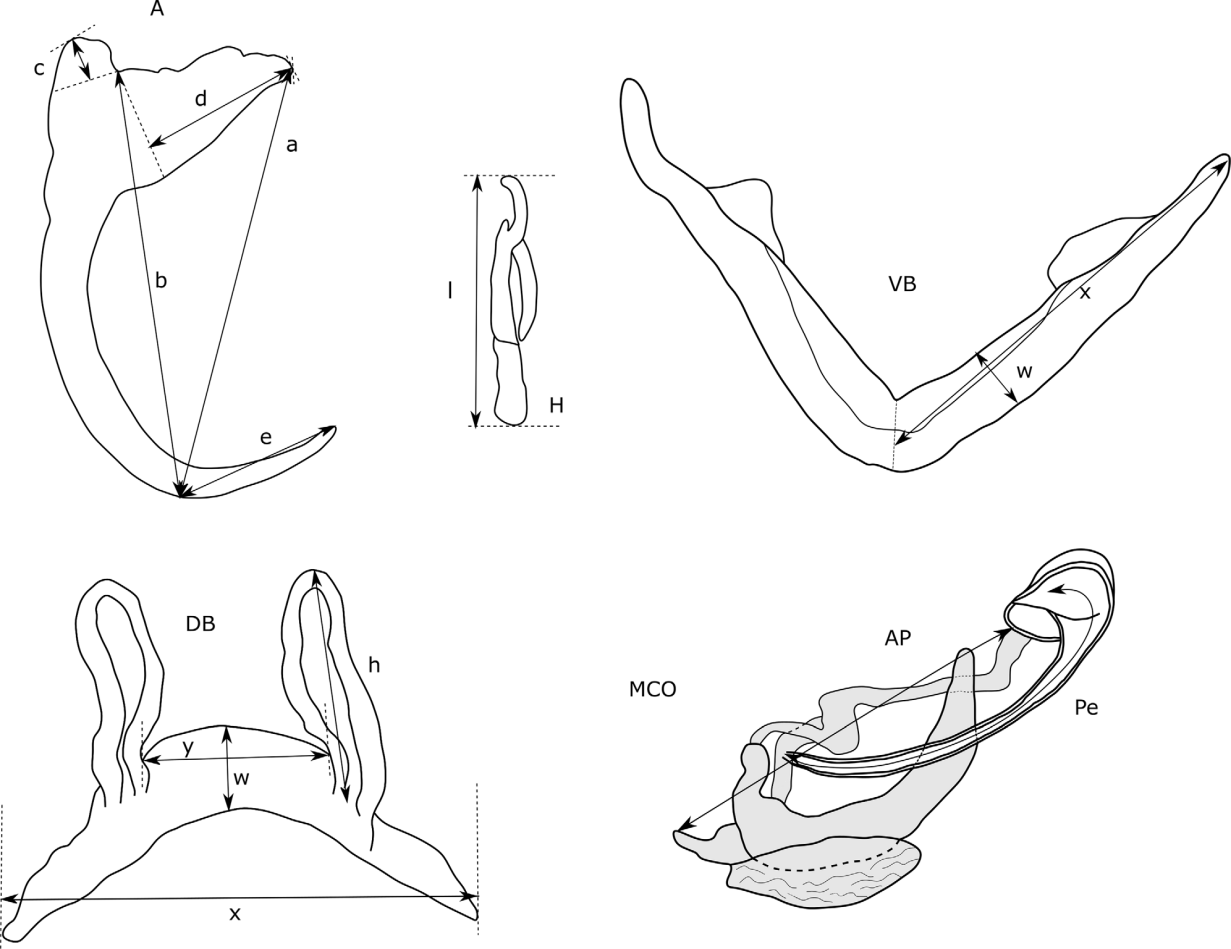
Measurements used for sclerotised structures of haptor and reproductive organs of *Cichlidogyrus* sp. VA, ventral anchor, DA, dorsal anchor: a, Total length, b, Length to notch, c, Outer root length, d, Inner root length, e, Point length; H, Hook length; VB, Ventral bar: x, Branch length, w, Branch width; DB, Dorsal bar: h, auricle length, w, maximum straight width, x, total length, y, distance between auricles; MCO, Male copulatory organ: AP, accessory piece straight length, Pe, copulatory tube curved length.

Species characterisations of dactylogyrid monogenean species are frequently based on the morphology of the sclerotised structures of the attachment and reproductive organs [95]. Therefore, parasites were grouped according to phenotypic characters in these structures. For an analysis of morphometric characters, we also took 29 different measurements of the hard parts of the haptor, the male copulatory organ (MCO), and the vagina (Fig. 2). The terminology was based on Pariselle *et al.* [78]. The marginal hooks are counted according to Llewellyn [60].

The monogeneans were identified to species level based on shapes and sizes of the sclerotised structures. However, to assess additional morphological variation in similar species, we conducted principal component analyses (PCA). Standard PCA approaches require a dataset without missing information, but often measurements of monogenean flatworms are incomplete due to the fragility of the worms and damage caused to the samples by the mounting process. Multiple methods have been proposed to address missing data in PCAs (see [119]). Here, we employ non-iterative partial least squares (NIPALS) with Gram–Schmidt orthogonalisation as implemented in the *R* package *nipals* v0.8 [130]. We obtained no DNA sequence data as the host specimens were initially fixed in formaldehyde solution, which leads to degradation of DNA molecules, and later transferred to 70% denatured ethanol (current storage).

### Phylogenetic position: maximum parsimony and machine learning

We used the morphometric data to infer the phylogenetic position of the new species of *Cichlidogyrus* based on the dataset published by Cruz-Laufer *et al.* [12]. Cruz-Laufer *et al.* [12] demonstrated that morphometric data of the attachment and reproductive organs can indicate phylogenetic relationships between species of *Cichlidogyrus*, albeit limited to certain measurements and groups of related species. Here, these data were reanalysed by expanding the parsimony and machine learning approaches to the new species found on chromidotilapiine cichlids.

First, phylogenetic positions were inferred based on the morphometric measurements (Fig. 2) and the morphological discrete characters for the reproductive organs suggested by Cruz-Laufer *et al.* [12] (Table 2). In some cases, we proposed new character states for the new species (Table 2). Phylogenetic inference was performed under maximum parsimony in TNT v1.5 [31, 32] with the latest genus-wide molecular phylogeny [12] used as a backbone (options *force* and *constrain*) to place the new species amongst their congeners with published DNA sequences. We applied extended implied weighting (option *xpiwe*) to reduce the impact of missing data [27] that were weighted artificially high in the original implied weighting method [28]. Furthermore, tree topologies were inferred for a range of values for the concavity constant *k* (20, 21, 23, 26, 30, 35, 41, 48, 56) to infer the most stable tree topology. We assigned each character a separate weight as recommended for continuous data [33]. As suggested by Mirande [68], we selected values of *k* that resulted in the highest distortion coefficient and subtree pruning and regrafting (SPR) distance on average compared to the other consensus trees. The final consensus tree was inferred from trees produced under the optimised *k* values. Tree searches involved rounds of tree fusing, sectorial searches, and tree drifting [29] under default settings with each round stopped after three hits of the same optimum. Gaps were treated as missing data. Branch support was estimated through symmetric resampling (probability of change: 0.33) and values expressed as differences in frequencies (GC: “Groups present/Contradicted”) as implied weighting can distort bootstrapping and jackknifing methods [30].

**Table 2:** List of character states of reproductive organs used for parsimony analysis. A new character state was used to capture the unique wing-like structure associated with the accessory pieces of *Cichlidogyrus ophioglossus* n. sp. and *C. gnomon* n. sp. (in bold: shape of accessory piece – o).

Character	Character states
Shape of copulatory tube	Straight: penis more or less straight with no strong arching, twisting, looping, or spiralling but can be slightly sinuous or arched. Straight, thick-walled: same as before but wall of penis present thickening. Arched: penis strongly arched in one direction, distal portion often held in position by accessory piece. Looped: penis draws a loop in the shape of a G. Large loop: penis draws large circle ending in distal portion of accessory piece. Spiralled: penis draws spiral in large radius. Spirally coiled: penis draws spiral in small radius in the shape of a helix.
Diameter of copulatory tube	Tubular: penis in the shape of a simple tube. Widened: penis widened. Bulbous: penis presents a bulbous portion (outside the basal bulb).
Shape of accessory piece	Simple: elongated accessory piece without additional structures mentioned in the other character states but species with more unique structures such as connecting stalks and caps are also included here. Furcated: accessory piece present one or more furcations. Distal hook: Accessory piece ends in a single distal hook. Distal flap: Accessory piece ends in a single distal flap. Gutter-like: Accessory piece in the shape of a gutter guiding the penis. Ribbon-like: Accessory piece is a flattened structure in the shape of a ribbon or drape. Spirally coiled: Accessory piece in the shape of simple helix. Looped: Accessory piece draws a loop in the shape of a G. Reduced: Accessory piece reduced to a thin, string-like structure or absent. Complex, S-shaped: massive, roughly S-shaped accessory piece that is frequently connected to the heel. The accessory piece has an extension or thickening at the first turn in the proximal half and frequently displays a folded back, straight and pointy, or hook-like distal end, or sometimes additional terminations resulting in a furcate ending with two or three digitations. However, the first turn is never V-shaped or knee-like such as in (l) and the hook-shaped termination is never sickle-like such as in (c). Complex, C-shaped: complicated roughly C-shaped accessory piece often with finger or hook-shaped outgrowths and marked heel. Two portions, V-shaped: accessory piece consists of two distinct portions shaped like a V with an expanded knee-like bend. Two portions, spiralling: accessory piece consists of two distinct portions, large spiral followed by non-spiralled distal portion. In two parts: accessory piece consists of two distinct, superimposed parts. **Complex with associated wing-like structure: accessory piece with multiple processes and associated wing-like structure.**
Shape of vagina	Non-sclerotised: Vagina not sclerotised. Tubular: Vagina in the shape of a simple tube. Bulbous: Vagina widened in at least one portion. Spiralled: tubular vagina that draws a spiral.

Second, species of *Cichlidogyrus* characterised here were placed in groups of related species of *Cichlidogyrus* reported by Cruz-Laufer *et al.* [12] using supervised machine learning (ML). Machine learning algorithms improve prediction accuracy through experience, *i.e.*, repetition. Here, we trained ML algorithms to classify specimens in species groups based on their morphology. Cruz-Laufer *et al.* [12] reported moderate performance of ML algorithms. However, their study only included one type of algorithm – support vector machines - and their algorithm was trained only on continuous morphometrics. In contrast, we applied three widely used ML algorithms including random forest (RF), support vector machines (SVM) with radial basis kernel function, and artificial neural networks (ANN) to all morphometric measurements combined as well as the discrete morphological characters of the reproductive organs proposed by Cruz-Laufer *et al.* [12]. This analysis was conducted in the *R* package *caret* (Kuhn, 2008) using the methods *rf* [58], *svmRadial* [67], and *nnet* [126]. Missing data were imputed through k-nearest neighbour imputation, and centred and scaled through the function *preProcess*. Tuning parameters were optimised through grid searches (Table 3) and ten-fold cross-validation with ten repetitions. Model performance was assessed through Cohen’s *κ* to account for the class imbalance in the data [57]. Following Landis and Koch [57], we considered *κ* < 0.2 *slight*, *κ* between 0.2 and 0.4 *fair*, *κ* between 0.4 and 0.6 *moderate*, *κ* between 0.6 and 0.8 *substantial*, and *κ* > 0.8 *almost perfect* agreement.

**Table 3:** Overview of range of values used for parameter tuning through grid search for different machine learning algorithms.

Algorithm	Parameters	Values for grid search
Support vector machines	*C*	2^−15^, 2^−13^, 2^−11^, 2^−9^, 2^−7^, 2^−5^, 2^−3^, 2^−1^, 2^1^, 2^3^
	*σ*	2^−5^, 2^−3^, 2^−1^, 2^1^, 2^3^, 2^5^, 2^7^, 2^9^, 2^11^, 2^13^, 2^15^
Artificial neural networks	Size	3, 5, 10, 20
	Decay	0.5, 0.1, 1E–2, 1E–3, 1E–4, 1E–5, 1E–6, 1E–7
Random Forest	Mtry	1, 2, 3, 4, 5, 6, 7, 8, 9, 10, 11, 12, 13, 14, 15, 16, 17, 18, 19, 20, 21, 22, 23, 24, 25

## Results

### Morphological examination

Of the 27 fish species examined, specimens of eight species and subspecies were infected with monogenean flatworms, including *Chromidotilapia elongata* Lamboj, 1999, *Chromidotilapia guntheri* (Sauvage, 1882), *Chromidotilapia guntheri loennbergii* (Trewavas, 1962), *Chromidotilapia kingsleyae* Boulenger, 1898, *Chromidotilapia linkei* Staeck, 1980, *Congochromis dimidiatus* (Pellegrin, 1900)*, Pelvicachromis roloffi* (Thys van den Audenaerde, 1968) and *Thysochromis ansorgii* (Boulenger, 1901). We found a total of 6, 69, 45, 135, 118, 2, 2, and 65 monogenean parasites, respectively. Eight species were found to be new to science, of which six belonging to *Cichlidogyrus* and two belonging to *Onchobdella*. Specimens of *Cichlidogyrus tilapiae* (Paperna, 1960) [73] and *Onchobdella krachii* Paperna, 1968 [72] were also found. Voucher material of *C. tilapiae* can be accessed at RMCA_VERMES_44395, RMCA_VERMES_44402, RMCA_VERMES_44445, RMCA_VERMES_44447, RMCA_VERMES_44453 44454, RMCA_VERMES_44459, RMCA_VERMES_44465, RMCA_VERMES_44492, RMCA_VERMES_44510, RMCA_VERMES_44514–44516, RMCA_VERMES_44549, HU XIX.2.17, HU XIX.2.20, MZH http://id.luomus.fi/KN.37266, MZH http://id.luomus.fi/KN.37274, SAMC-A095109–A095110, and MNHN HEL1912–1913. Species descriptions and characterisations are presented in the following. Note that the authors of the new species are different from the authors of the article [according to Article 50 of the International Code of Zoological Nomenclature (ICZN)]. Infection parameters can be found in Table 1. Symbiotypes and symbioparatypes are given as follows: RMCA accession number (specimen IDs).

### *Cichlidogyrus ataikputu* Moons, Kmentová, Pariselle, Vanhove & Cruz-Laufer n. sp.

urn:lsid:zoobank.org:act:F82C828C-FD7B-447F-B928-C208EA48EF41

Type host*: Chromidotilapia guntheri* (Sauvage, 1882).

Additional host*: Chromidotilapia linkei* Staeck, 1980.

Type locality: New Calabar river, Akpor, Nigeria; 4.87, 6.90; 01/12/1990 on type host.

Additional locality: Lake Barombi-Kotto, Cameroon on *Chromidotilapia guntheri* and road Yabassi-Yingui, Cameroon on *Chromidotilapia linkei.*

Material: 15 whole-mounted specimens fixed in Hoyer’s medium.

Holotype: RMCA_VERMES_44462.

Paratypes: RMCA_VERMES_44411, RMCA_VERMES_44413, RMCA_VERMES_44415, RMCA_VERMES_44458, RMCA_VERMES_444460–44461, HU 847–848, HU 853, MZH http://id.luomus.fi/KN.37261–http://id.luomus.fi/KN.37262, SAMC-A095116–A095117, MNHN HEL1914–1917.

Symbiotype: RMCA_Vert_1991.010.P.0542-0582 (578).

Symbioparatype: RMCA_Vert_1991.010.P.0542-0582 (576, 577, 581, 582); RMCA_Vert_1973.005.P.4955-4978 (CGL9, CGL16, CGL20); RMCA_Vert_1992.144.P.0250-0261 (B, C).

Infection site: gills.

Etymology: The species epithet “*ataikputu*” correctly spelled “ata ikputu” is Igbo, a language spoken in the area where the holotype was sampled. “ata” translates to “consumes”, whereas “ikputu” refers to Gunther’s mouthbrooder (*Chromidotilapia guntheri*) [123].

Note: The authors of the new taxa are different from the authors of this paper: Article 50.1 and Recommendation 50A of the International Code of Zoological Nomenclature [38].

#### Description (Table 4, Fig. 3)

Two pairs of anchors. Ventral anchor with more developed inner root than outer root and deep indentation. Dorsal anchors with well-developed inner root. Sturdy ventral transverse bar V-shaped with small membranous attachment at base of branches. Dorsal transverse bar with thick midsection and long and slender auricles. Marginal hooks seven pairs, all approximately same size except for pair 2, which is smaller. Male copulatory organ (MCO) consists of copulatory tube and accessory piece. Copulatory tube has broad base and becomes slender and curved towards distal end, where it is guided by sheath-like portion of accessory piece. Accessory piece is as broad as copulatory tube and is attached to base of copulatory tube. At distal end, accessory piece bends at a 90° angle and follows copulatory tube terminating in two small pointy protuberances. Accessory piece folds halfway and guides copulatory tube. No heel present. No sclerotised vagina observed.

**Figure 3:**
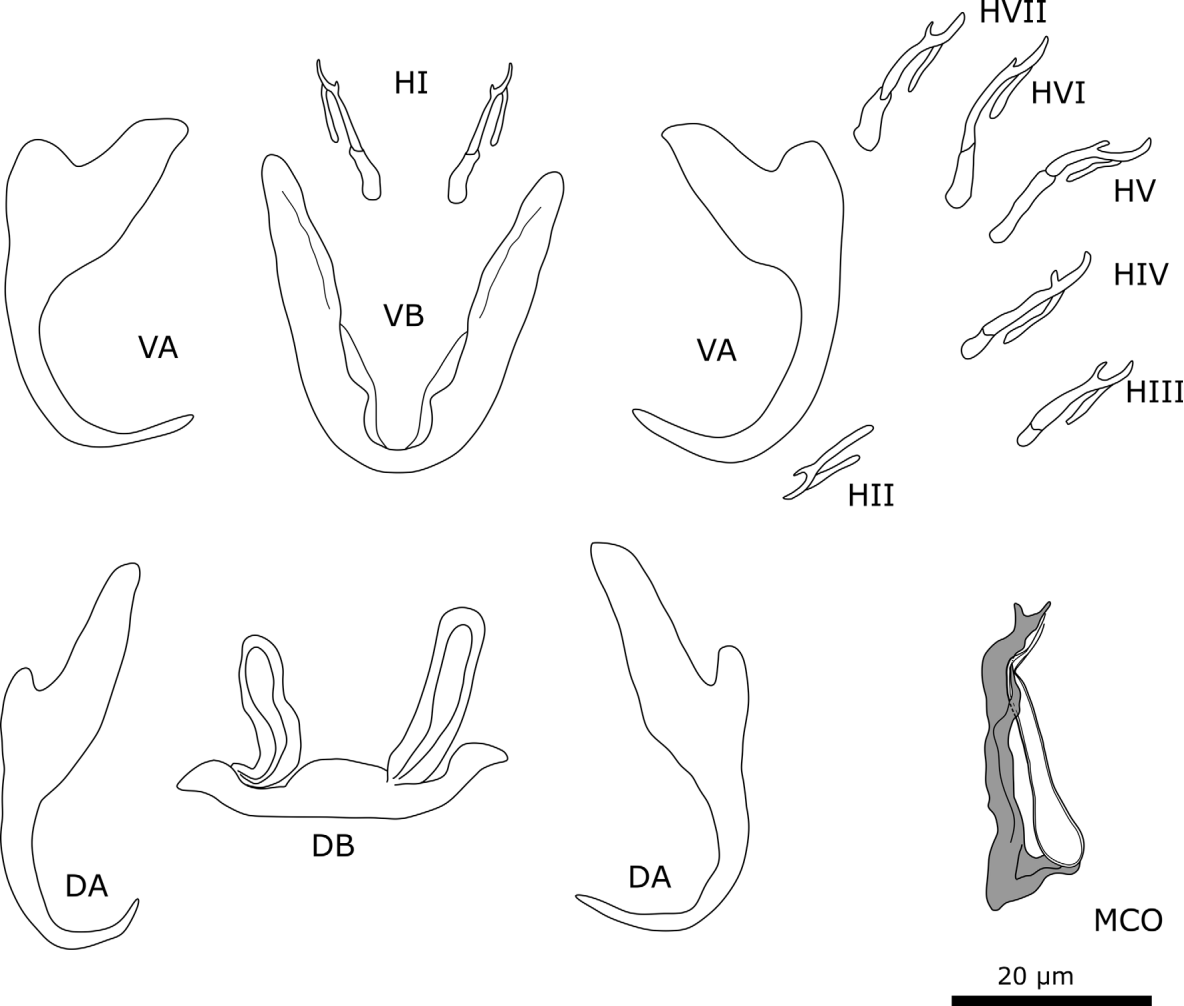
Sclerotised structures of *Cichlidogyrus ataikputu* n. sp. Abbreviations: HI-HVII, hooks; VA, ventral anchor; VB, ventral transverse bar; DA, dorsal anchor; DB, dorsal transverse bar; MCO, male copulatory organ.

**Table 4:** Morphometrics of species of *Cichlidogyrus* infecting chromidotilapiines cichlids. min–max, minimum and maximum value; SD, standard deviation; *n*, sample size.

Measure	*Cichlidogyrus ataikputu* n. sp.				*Cichlidogyrus dibangoi* n. sp.				*Cichlidogyrus ophioglossus* n. sp.				*Cichlidogyrus gnomon* n. sp.				*Cichlidogyrus* sp. “*Pelvicachromis roloffi*”				*Cichlidogyrus thysochromis* n. sp.				*Cichlidogyrus tilapiae* ex *Chromidotilapia* spp.				*Cichlidogyrus tshuapa* n. sp.			
	mean	min–max	SD	*n*	mean	min–max	SD	*n*	mean	min–max	SD	*n*	mean	min–max	SD	*n*	mean	min–max	SD	*n*	mean	min–max	SD	*n*	mean	min–max	SD	*n*	mean	min–max	SD	*n*
DAa	40.9	36.5–42.9	1.9	10	42.4	42.3–42.4	0.1	2	38.5	26.3–42.1	2.8	47	41.6	40.9–42.3	1.0	2	–	–	–	0	21.7	–	–	1	39.1	31.6–43.8	3.3	12	43.9	43.4–44.4	0.7	2
DAb	25.9	25.0–27.8	1.0	9	25.8	24.8–26.8	1.4	2	29.9	22.9–37.1	2.3	47	29.6	29.5–29.7	0.1	2	–	–	–	0	18.4	–	–	1	25.7	23.6–29.7	1.9	12	22.4	21.1–23.6	1.8	2
DAc	4.7	3.0–7.0	1.1	11	6.7	6.0–7.7	0.9	3	4.5	1.4–6.9	1.1	48	7.5	7.3–7.7	0.3	2	–	–	–	0	12.4	–	–	1	5.7	2.6–9.2	1.6	17	13.3	12.5–14.1	1.1	2
DAd	19.1	15.3–26.8	3.3	12	21.3	19.9–22.9	1.5	3	11.8	6.2–18.3	2.2	48	15.0	14.9–15.0	0.1	2	–	–	–	0	4.5	–	–	1	20.0	13.1–28.0	3.2	18	25.4	25.3–25.5	0.1	2
DAe	10.7	9.5–11.4	0.7	8	11.2	10.4–11.9	1.1	2	9.2	4.0–11.7	1.2	47	9.6	8.1–11.0	2.1	2	–	–	–	0	10.0	–	–	1	11.0	10.1–13.5	1.0	11	7.4	–	–	1
DBh	19.8	11.1–26.1	4.0	11	22.2	20.3–24.5	2.1	3	16.7	11.7–23.1	2.1	43	15.0	–	–	1	–	–	–	0	12.3	–	–	1	20.6	15.8–26.3	2.9	15	–	–	–	0
DBw	5.7	3.6–6.9	1.0	11	6.3	5.9–6.7	0.4	3	14.1	9.6–18.6	1.9	44	13.7	–	–	1	–	–	–	0	5	–	–	1	6.1	4.9–7.6	0.8	18	5.5	4.9–6.1	0.8	2
DBx	19.2	17.1–20.9	1.3	10	19.4	18–21.4	1.8	3	22.0	14.5–27.7	2.5	50	22.0	21.5–22.4	0.6	2	–	–	–	0	38.2	–	–	1	18.2	12.2–20.6	2.1	18	22.4	20.2–24.5	3.0	2
DBy	11.0	6.2–19.5	3.7	10	11.7	10.8–12.5	0.9	3	5.6	3.6–7.1	0.7	51	5.6	5.1–6.6	0.9	3	–	–	–	0	10.5	–	–	1	9.6	6.3–12.9	2.0	17	13.0	–	–	1
VAa	33.2	27.8–37.2	2.7	10	33.6	32.2–35	1.4	3	37.5	29.7–46	3.2	50	38.3	36.4–40.2	2.7	2	–	–	–	0	33.2	–	–	1	32.8	27.6–36.5	2.5	16	36.3	35.8–36.7	0.6	2
VAb	28.2	25.7–30.2	1.4	10	27.1	25.7–29.6	2.2	3	34.4	29.6–38.3	1.9	50	33.4	31.4–35.3	2.8	2	–	–	–	0	30.1	–	–	1	28.0	25.1–32.3	1.9	16	28.7	28.7–28.7	0.0	2
VAc	5.9	4–8.1	1.6	11	6.1	4.9–6.8	1.0	3	3.7	1.4–6.2	1.0	50	5.2	5.0–5.4	0.3	2	–	–	–	0	4.5	–	–	1	5.9	3.4–8.3	1.5	17	10.7	–	–	1
VAd	12.8	9.4–15.8	2.2	11	14.8	12.6–16.3	1.9	3	10.4	7.9–13.9	1.3	50	11.6	11.1–12	0.6	2	–	–	–	0	11.7	–	–	1	13.7	11–17.3	1.8	17	15.3	14.5–16.1	1.1	2
VAe	11.8	8.8–14.6	1.7	11	12.3	11.7–13.4	1.0	3	11.3	7.8–14.3	1.4	51	12.8	12.2–13.3	0.8	2	–	–	–	0	8.9	–	–	1	11.8	8.6–14	1.4	16	12.7	12.5–12.8	0.2	2
VBw	5.4	4.0–6.6	0.9	8	5.9	5.3–6.3	0.6	3	4.8	3.0–6.6	0.9	49	5.3	5.1–5.4	0.2	2	–	–	–	0	4.7	–	–	1	5.4	4.4–6.6	0.7	15	5.3	5–5.6	0.4	2
VBx	33.7	27.2–41.6	5.0	7	31.7	29.6–33.3	1.9	3	34.7	29.2–42.2	2.9	46	35.2	33.9–36.5	1.8	2	–	–	–	0	34.9	–	–	1	33.9	28–40.2	3.8	14	40.4	40–40.7	0.5	2
HI	16.0	15.1–16.8	0.6	11	13.7	13.7–13.7	–	1	15.4	12.6–18.6	1.5	37	12.6	10.4–15.2	2.4	3	–	–	–	0	28.7	–	–	1	16.5	14.4–19.1	1.3	13	27.9	27.1–28.6	1.1	2
HIshaft	4.3	3.6–5.1	0.5	10	–	–	–	0	–	–	–	0	–	–	–	0	–	–	–	0	14	–	–	1	4.4	3.7–5.8	0.6	13	17.0	15.7–18.3	1.8	2
HII	12.2	11.2–13.6	1.2	3	–	–	–	0	14.1	11.2–19.6	3.6	6	–	–	–	0	–	–	–	0	9.7	–	–	1	12.1	10.8–13.8	1.1	5	10.0	–	–	1
HIII	19.0	18.5–19.5	0.7	2	–	–	–	0	17.0	11.4–20	3.2	7	18.7	–	–	1	–	–	–	0	15.3	–	–	1	17.8	16.4–20.2	1.5	5	24.6	–	–	1
HIV	15.8	14–17.4	1.7	3	–	–	–	0	15.6	13.3–19.7	2.3	7	14.9	–	–	1	–	–	–	0	16.3	–	–	1	15.0	13.9–16	0.9	6	16.0	14–17.9	2.8	2
HV	19.2	15.1–21.7	2.6	7	20.9	17.9–23.8	4.2	2	20.1	16.1–23.1	2.0	22	14.8	–	–	1	–	–	–	0	–	–	–	0	19.9	16.1–22.4	1.7	10	27.7	26.7–28.7	1.4	2
HVI	19.4	8.6–22.9	4.1	9	21.0	20.2–21.7	1.1	2	19.7	17.8–22.1	1.0	24	15.9	13–18.7	4.0	2	–	–	–	0	15.6	–	–	1	19.1	17.2–21.4	1.4	9	26.3	25.3–27.3	1.4	2
HVII	17.6	16–19.1	1.2	8	20.4	20–20.7	0.5	2	17.4	14.1–20.3	1.6	24	17.8	17.3–18.2	0.6	2	–	–	–	0	16.5	–	–	1	17.6	15.9–19.7	1.2	8	22.9	21.8–23.9	1.5	2
Pe	25.0	18–34.3	4.1	14	24.4	18.4–29.4	5.6	3	29.2	19–39.5	4.3	52	26.1	24.3–30.3	2.8	4	28.7	–	–	1	–	–	–	0	27.5	21.4–36.8	4.7	19	22.7	22–23.3	0.9	2
AP	27.2	18.3–37.3	5.8	15	28.5	22.4–31.6	5.3	3	28.5	18.8–45.2	5.4	52	23.0	17.3–26.6	4.1	4	29.3	–	–	1	–	–	–	0	26.8	22.9–33.6	3.2	18	27.9	26.5–29.3	2.0	2

#### Remarks

The specimens show typical features of species of *Cichlidogyrus*, *i.e.*, (i) two pairs of anchors (one ventral and one dorsal), two transverse bars (V-shaped ventral bar, dorsal bar with two auricles); (ii) seven pairs of marginal hooks; (iii) an MCO consisting of a copulatory tube and generally an accessory piece; and (iv) a vagina, which can be sclerotised [73, 79]. *Cichlidogyrus ataikputu* n. sp. presents similarities with *Cichlidogyrus tilapiae*. The dorsal anchors of the two species are similar in having a well-developed inner root and a reduced outer root. The shapes of the dorsal bars are also similar, as are the lengths of the auricles in *C*. *ataikputu* n. sp. (11.1–26.1 μm) and *C. tilapiae* (23–34 μm) according to Rindoria *et al.* [102] and the original measurements by Paperna [73]: 9–19 μm. *Cichlidogyrus ataikputu* n. sp. also resembles *Cichlidogyrus dibangoi* n. sp., also described in the present study (see below). The average dorsal bar auricle length is larger in *C*. *dibangoi* n. sp. The hooks of *C. dibangoi* n. sp. and *C. ataikputu* n. sp*.* are very similar in morphology and size. At the distal end, the accessory piece of *C. dibangoi* n. sp. encompasses the copulatory tube like a sheath from one side. This sheath-like portion of the accessory piece is also seen in *C*. *ataikputu* n. sp. but is shorter than in *C. dibangoi* n. sp. The end of the accessory piece shows two small protuberances, whereas in *C. dibangoi* n. sp., the end is hook-shaped. The morphology of the copulatory tube is similar in *C. dibangoi* n. sp. and *C*. *ataikputu* n. sp., in having a bulbous base followed by a long slender tube. Yet the tube curves at the distal end in *C*. *ataikputu* n. sp., whereas in *C. dibangoi* n. sp. the tube is straight, and no heel is present unlike in *C. dibangoi* n. sp.

### *Cichlidogyrus dibangoi* Moons, Kmentová, Pariselle, Vanhove & Cruz-Laufer n. sp.

urn:lsid:zoobank.org:act:385363DE-F900-439E-94A1-A175674EFB00

Type host: *Chromidotilapia guntheri* (Sauvage, 1882).

Additional host: *Chromidotilapia linkei* Staeck, 1980.

Type locality: Lake Barombi-Kotto, Cameroon; 4.47, 9.25; 24/10/1966.

Additional locality: road from Yabassi to Yingui, Cameroon; on *Chromidotilapia linkei.*

Material: 3 whole-mounted specimens fixed in Hoyer’s medium.

Holotype: RMCA_VERMES_44504.

Paratypes: RMCA_VERMES_44551, RMCA_VERMES_44554.

Symbiotype: RMCA_Vert_1973.005. P.4955-4978 (CGL16).

Symbioparatype: RMCA_Vert_1992.144.P.0250-0261 (B).

Infection site: gills.

Etymology: The species epithet “*dibangoi”* honours Manu Dibango, a famous saxophonist and singer-songwriter from Cameroon, who incorporated Jazz and traditional Cameroonian elements into his music.

Note: The authors of the new taxa are different from the authors of this paper: Article 50.1 and Recommendation 50A of the International Code of Zoological Nomenclature [38].

#### Description (Table 4, Fig. 4)

Two pairs of anchors. Ventral anchors with more developed inner root than outer root. Dorsal anchors have well-developed inner root and outer root about the same size as outer root of ventral anchor. Sturdy ventral transverse bar V-shaped with membranous attachment towards distal end of branches. Dorsal transverse bar with thick middle section and elongated slender auricles. Seven pairs of marginal hooks have approximately the same size, except for pair 2 which is smaller; measurements of pair 3 and 4 could not be assigned due to distortions of material during mounting process. MCO consists of copulatory tube, accessory piece, and small heel. Copulatory tube is broad at the base, narrows towards distal end with terminal opening. Accessory piece is attached to base of copulatory tube. Proximal part of accessory piece folds towards copulatory tube. Distally, accessory piece widens, then narrows again towards hook-shaped distal end. Accessory piece folds at mid-portion of copulatory tube. No sclerotised vagina observed.

**Figure 4:**
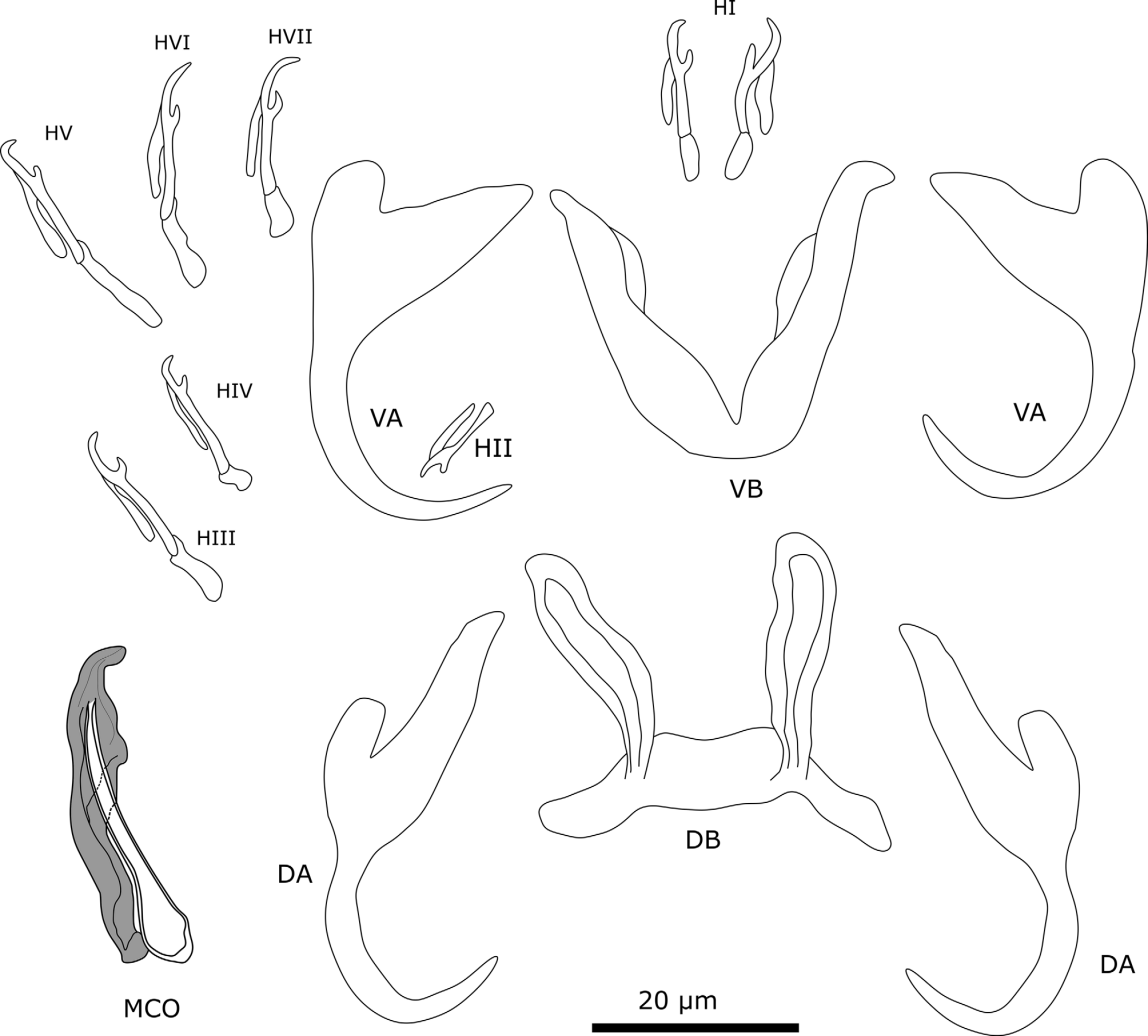
Sclerotised structures of *Cichlidogyrus dibangoi* n. sp. Abbreviations: HI-HVII, hooks; VA, ventral anchor; VB, ventral transverse bar; DA, dorsal anchor; DB, dorsal transverse bar; MCO, male copulatory organ.

#### Remarks

All specimens show diagnostic features of species of *Cichlidogyrus* (see “Remarks” *C. ataikputu* n. sp.). *Cichlidogyrus dibangoi* n. sp. resembles *C. tilapiae*, which infects a wide array of cichlid and non-cichlid hosts [12], and *Cichlidogyrus ataikputu* n. sp. The ventral anchors are morphologically similar to *C. tilapiae* and *C. ataikputu* n. sp. in their size and lengths of their roots. Furthermore, the three species have a dorsal bar that is similar in size with long slender auricles. The auricles are slightly longer in *C. dibangoi* n. sp. (20.3–24.5 μm) than in *C. tilapiae* (9–19 μm) described by Paperna [73], but not longer than *C. tilapiae* (23–34 μm) reported by Rindoria *et al.* [102]. The differences in sizes might be explained by different mounting media [17], but also by an adaptation to different host species or geographical variation, *e.g.*, *Oreochromis niloticus* (Linnaeus, 1758) and *Sarotherodon galilaeus* (Linnaeus, 1758) in Dor, Israel [73]; and *O. leucostictus* (Trewavas, 1933) and *O. niloticus* in Lake Naivasha, Kenya [102]. The MCO resembles that of *C. tilapiae*. The accessory piece of both species widens distally and terminates in a hook-like structure although, in *C. dibangoi* n. sp., the structure encloses the copulatory tube in the distal half, which is not the case in *C. tilapiae*. In *C*. *dibangoi* n. sp., the accessory piece guides the distal portion of the copulatory tube. The copulatory tube of *C*. *dibangoi* n. sp. is also associated with a small heel, which is absent in *C. tilapiae*.

### *Cichlidogyrus ophioglossus* Moons, Kmentová, Pariselle, Vanhove & Cruz-Laufer n. sp.

urn:lsid:zoobank.org:act:5086857C-1862-4C5D-B716-141BCF7D3650

Type host: *Chromidotilapia kingsleyae* Boulenger, 1898 (Perciformes: Cichlidae).

Additional host: *Chromidotilapia elongata* Lamboj, 1999 (Perciformes: Cichlidae).

Type locality: small stream, affluent of Moukalaba, Nyanga basin, Gabon; −02.78, 10.77; 19/09/2001; on type host.

Additional locality: Congo Republic; −4.28, 12.45; on *Chromidotilapia elongata.*

Material: 76 whole-mounted specimens fixed in Hoyer’s medium.

Holotype: RMCA_VERMES_44527.

Paratypes: RMCA_VERMES_44369, RMCA_VERMES_44517–44526, RMCA_VERMES_44528–44547, HU 842, HU 849–852, MZH http://id.luomus.fi/KN.37267–http://id.luomus.fi/KN.37271, SAMC-A095118–A095122, MNHN HEL1918–1922.

Symbiotype: RMCA_Vert_2002.006.P.2722-2768 (D).

Symbioparatype: RMCA_Vert_1991.068.P.2121-2125 (LA).

Infection site: gills.

Etymology: The species epithet “*ophioglossus”* is derived from the Greek word *ophis = snake* and *glossa = tongue*, and refers to the morphology of the accessory piece in the male copulatory organ that resembles a forked tongue of a snake.

Note: The authors of the new taxa are different from the authors of this paper: Article 50.1 and Recommendation 50A of the International Code of Zoological Nomenclature [38].

#### Description (Table 4, Fig. 5)

Two pairs of anchors. Ventral anchors with reduced outer root, inner root more developed. Indentation between roots relatively shallow. Dorsal anchors about the same size as ventral anchors. Inner root of dorsal anchor well-developed and outer root reduced. Between inner and outer root, anchor shows small bulge. Ventral transverse bar V-shaped with triangular membranous attachments at distal half of branches. Dorsal transverse bar has thick midsection with two pronounced auricles. Seven pairs of marginal hooks; pairs 1, 3, 4, 5, 6, and 7 with approximately the same length; pair 2 small. Secondary shaft shorter than pair 1 and 4. MCO consists of copulatory tube and accessory piece. Copulatory tube long and slightly curved, narrowing distally, with distal opening; basal bulb broad with heel attached. Accessory piece consisting of two parts, large distal portion and proximal connecting piece. Large portion is slightly curved, with broadened section partly engulfing copulatory tube. Distal end of large portion of accessory piece bifurcating, one end protrudes in bulbous end, other end forms hook with wing-shaped, serrated structure. Connecting piece (string-like structure, see below) is attached at base of copulatory tube, bifurcating at end of the copulatory tube, connecting with bulbous end of large portion of accessory piece. Considerable variation in MCO morphology in specimens found on same host individuals (see Fig. 6). String-like structure attaches to end of base of copulatory tube. This attachment point is similar in all individuals. However, flattening of specimens during mounting process results in different appearances (see Figs. 6A, 6B). In some individuals, string-like structure draws a loop or is curved. In other individuals, this structure is concealed or broken. Hence, shape of the structure was not always observed. In these cases, connecting portion is concealed by large portion of accessory piece, which might create the illusion that large portion is directly connected with copulatory tube where string-like structure would attach (see Fig. 6C). Furthermore, wing-shaped structure might appear larger and more open in these individuals (Fig. 6C); whereas usually this structure mostly (or partially) overlaps with large portion of accessory piece. Sclerotised vagina is tubiform, drawing a U-turn.

**Figure 5:**
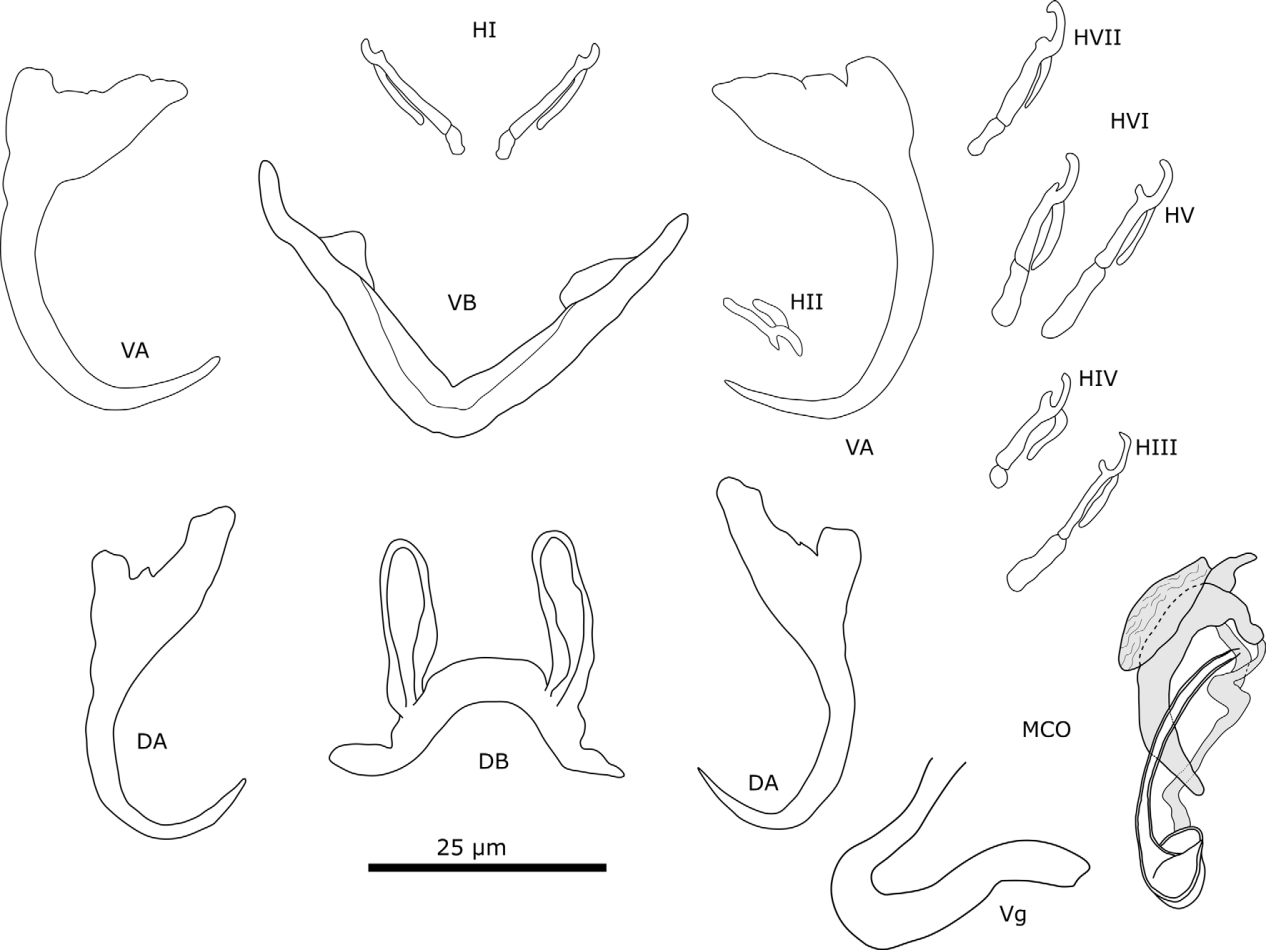
Sclerotised structures of *Cichlidogyrus ophioglossus* n. sp. Abbreviations: HI-HVII, hooks; VA, ventral anchor; VB, ventral transverse bar; DA, dorsal anchor; DB, dorsal transverse bar; MCO, male copulatory organ; Vg, vagina.

**Figure 6:**
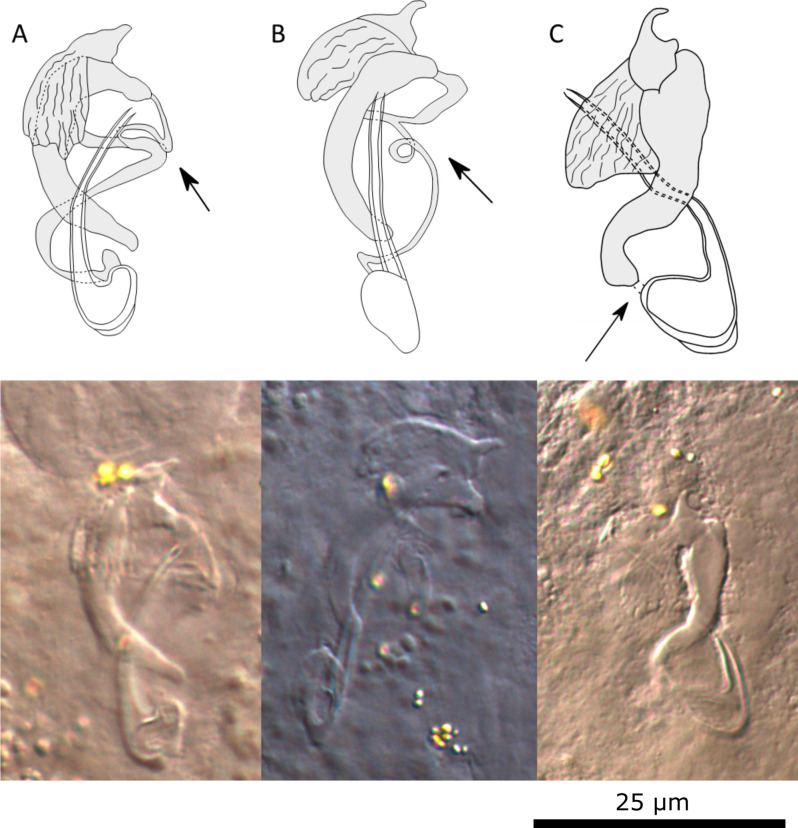
Drawings and microscopic pictures of the male copulatory organs of multiple individuals of *C. ophioglossus* n. sp. Arrows indicating the variation seen in different specimens.

#### Remarks

All specimens show diagnostic features of species of *Cichlidogyrus* (see “Remarks” *C. ataikputu* n. sp.). *Cichlidogyrus ophioglossus* n. sp. resembles *C. acerbus* Dossou, 1982 [14], *C. fontanai* Pariselle & Euzet, 1997 [81], *C. lagoonaris* Paperna, 1969 [74], and *C. nageus* Řehulková, Mendlová & Šimková, 2013 [100]; all infecting *Sarotherodon* species [14, 74, 81, 100]. *Cichlidogyrus acerbus, C. fontanai*, *C. lagoonaris,* and *C. nageus* share similarities with *C. ophioglossus* n. sp. in the morphology of the ventral bar. The species have a V-shaped bar with membranous triangles attached at the midsection. The dorsal anchors are also similar in having a well-developed inner root. The ventral anchors of *C. acerbus*, *C. fontanai, C. lagoonaris,* and *C. nageus* present distinct roots, with the inner root being more developed than the outer root, while *C. ophioglossus* n. sp. has no distinct roots. The dorsal bar has well-developed auricles in *C. ophioglossus* n. sp. and the other species, yet the midsection of the dorsal bar is thicker in *C. fontanai* (12 μm) and *C. nageus* (8 μm) [81, 100] than in *C. ophioglossus* n. sp. (5.6 μm). The copulatory tube in *C. ophioglossus* n. sp. is similar to *C. fontanai*, *C. lagoonaris*, and *C. nageus*, which also have a slightly curved copulatory tube with a broad base. The size of the heel in *C. ophioglossus* n. sp. is as small as observed in *C. fontanai*. The accessory piece of *C. fontanai* is bifurcated at the distal end, which is also seen in *C. ophioglossus* n. sp. Furthermore, a smaller portion of the accessory piece is also observed in *C. nageus*. This part is connected to the broad base of the copulatory tube, as in *C. ophioglossus* n. sp. However, the small portion is string-like in *C. ophioglossus* n. sp., but broader and more finger-like in *C. nageus.* The larger portion of the accessory piece ends in three processes, of which one is hook-shaped, also seen in *C. ophioglossus* n. sp. but here a wing-shaped serrated structure is attached to it. No wing-like serrated structure has been reported in any species of *Cichlidogyrus* to date.

### *Cichlidogyrus gnomon* Moons, Kmentová, Pariselle, Vanhove & Cruz-Laufer n. sp.

urn:lsid:zoobank.org:act:19F5F9BF-4454-4165-808D-14D910A06F54

Type host: *Chromidotilapia elongata* Lamboj, 1999 (Perciformes: Cichlidae)

Type locality: Mavemba river, tributary of Loukoula on the right bank, 2 km downstream from Mpounga, Republic of the Congo; −4.28, 12.45; 30/07/1991.

Material: 4 whole-mounted specimens fixed in Hoyer’s medium.

Holotype: RMCA_VERMES_44367.

Paratypes: RMCA_VERMES_44366, RMCA_VERMES_44368, SAMC-A095104.

Symbiotype: RMCA_Vert_1991.068.P.2121-2125 (LA).

Infection site: gills.

Etymology: The species epithet “*gnomon*” refers to the part of a sundial that casts a shadow. The term is commonly used to refer to an L-shape in geometry. Here, “*gnomon*” refers to the L-shaped accessory piece of the male copulatory organ.

Note: The authors of the new taxa are different from the authors of this paper: Article 50.1 and Recommendation 50A of the International Code of Zoological Nomenclature [38].

#### Description (Table 4, Fig. 7)

Two pairs of anchors. Ventral anchor with reduced outer root, inner root more developed. Dorsal anchor approximately the same size as the ventral anchor. Outer root of dorsal anchor reduced, slightly larger than the outer root of the ventral anchor. Inner root more developed and larger than the inner root of the ventral anchor. Ventral transverse bar V-shaped with triangular membranous attachments along distal half of branches. Dorsal transverse bar has a thick midsection with auricles. Auricles are drop-shaped. Most likely seven pairs of marginal hooks like all congeners, but pair 2 was not observed due to the poorly preserved specimens. Pairs 1 and 3–7 approximately the same length. Secondary shaft of pairs 1 and 4 shorter. Male copulatory organ consists of a copulatory tube and an accessory piece. Distal opening of the copulatory tube slightly curved. Copulatory tube narrows towards the distal end and has a broad basal bulb with a small heel. Accessory piece bends in the middle portion and connects to the base of the copulatory tube at two points. Distal end of the accessory piece splits and forms a long and a short projection, each with a bulbous portion. Shorter protrusion connected to a plate. This plate has a hook-like projection, a small bulge at the distal end, and a drop-like projection at the proximal end. No sclerotised vagina observed.

**Figure 7:**
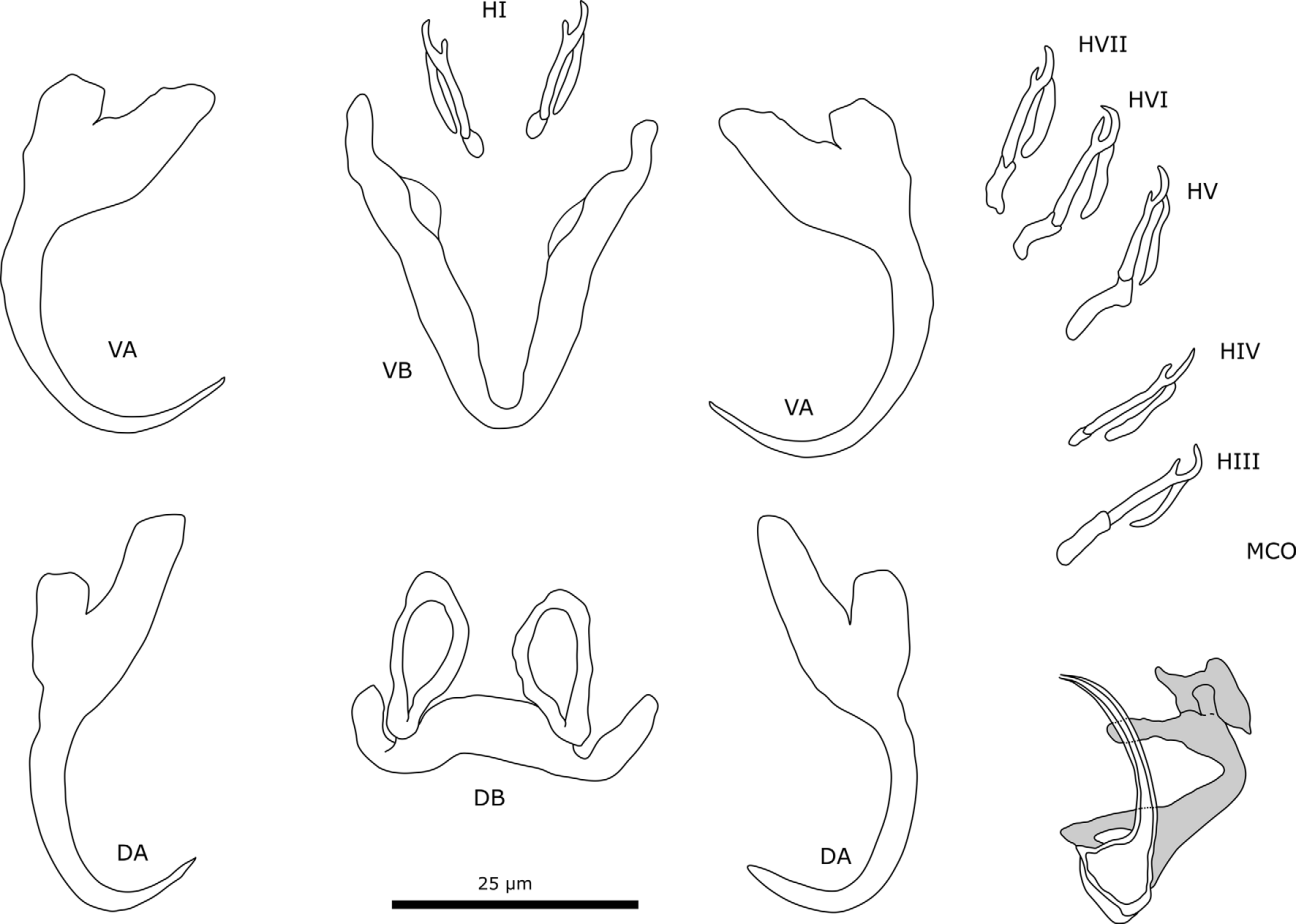
Sclerotised structures of *Cichlidogyrus gnomon* n. sp. Abbreviations: HI-HVII, hooks; VA, ventral anchor; VB, ventral transverse bar; DA, dorsal anchor; DB, dorsal transverse bar; MCO, male copulatory organ.

#### Remarks

All specimens show diagnostic features of species of *Cichlidogyrus* (see “Remarks” *C. ataikputu* n. sp.). *Cichlidogyrus gnomon* n. sp. resembles *C. fontanai* [infecting *Sarotherodon occidentalis* (Daget, 1962) in the Bourouma River (Guinea)]; in the same way, it resembles *C. ophioglossus* n. sp. The protrusions at the distal end of the accessory piece are hook-like in *C. fontanai* but bulbous in *C. gnomon* n. sp. The outer roots of the dorsal anchor are larger in *C*. *gnomon* n. sp. (7.5 μm compared to 2.0 μm in *C. fontanai*); the inner roots of the ventral anchor (15.0 μm) are slightly larger than the inner root of the dorsal anchor (11.6 μm). In *C. fontanai*, the size difference of the inner roots of dorsal and ventral anchors is less pronounced (10 μm and 13 μm). The dorsal transverse bars are similarly shaped, but the dorsal bar is generally larger in *C. fontanai* (DBx = 34 μm compared to 22 μm in *C. gnomon* n. sp.). The dorsal bar of *C. muterezii* Pariselle & Vanhove, 2015 [124] resembles *C*. *gnomon* n. sp., but the midsection is thinner in *C. muterezii* (6.4 μm) than in the former species (13.7 μm). *Cichlidogyrus gnomon* n. sp. resembles *C. ophioglossus* n. sp. in a number of characters. First, the sizes of the ventral and dorsal anchors are similar; morphologically, the species differ in the ventral anchors as the incision between the roots is more pronounced in *C. gnomon* n. sp. than in *C. ophioglossus* n. sp. In *C. ophioglossus* n. sp., the accessory piece attaches to the base of the copulatory tube with a small string-like extension, whereas in *C. gnomon* n. sp., the accessory piece attaches to the base of the copulatory tube directly. The accessory piece bifurcates at the distal end for both species but in *C. gnomon* n. sp. this results in two bulbous protuberances. An additional plate-like structure is connected to one of these protuberances, which unlike the wing-shaped structure in *C. ophioglossus* n. sp., is not serrated.

### *Cichlidogyrus tshuapa* Moons, Kmentová, Pariselle, Vanhove & Cruz-Laufer n. sp.

urn:lsid:zoobank.org:act:C1EDD96A-C2FD-4E30-BFAC-AE7183F6F0FB

Type host: *Congochromis dimidiatus* (Pellegrin, 1900).

Type locality: Boende, Tshuapa Province, Democratic Republic of the Congo; 0.23, 20.83; 29/09/1969.

Material: 2 whole-mounted specimens fixed in Hoyer’s medium.

Holotype: RMCA_VERMES_44385.

Paratype: RMCA_VERMES_44386.

Symbiotype: RMCA_Vert_P.174947-174968 (967).

Infection site: gills.

Etymology: The species epithet refers to the province Tshuapa in the Democratic Republic of Congo, where the species was found.

Note: The authors of the new taxa are different from the authors of this paper: Article 50.1 and Recommendation 50A of the International Code of Zoological Nomenclature [38].

#### Description (Table 4, Fig. 8)

Two pairs of anchors. Ventral anchors with well-developed inner and outer root. Dorsal anchor also with well-developed inner and outer root with outer root being about half the length of inner root. Both dorsal and ventral anchors have deep indentations between the roots. Ventral transverse bar V-shaped with membranous attachments over most of the length of branches. Dorsal transverse bar with thickened midsection and auricles. Seven pairs of marginal hooks. First pair is large (“standardised length” larger than 1.7 following Pariselle & Euzet [79]) with long and broad secondary shafts. Pairs 3–7 approximately the same length. Pair 2 is the smallest. The male copulatory organ consists of copulatory tube, accessory piece, and heel. The copulatory tube has broad middle section and narrows at distal end. S-shaped accessory piece is attached to heel, which is small. Distal portion is positioned parallel to copulatory tube. Sclerotised vagina is pear-shaped with a tubiform sinuous extension.

**Figure 8:**
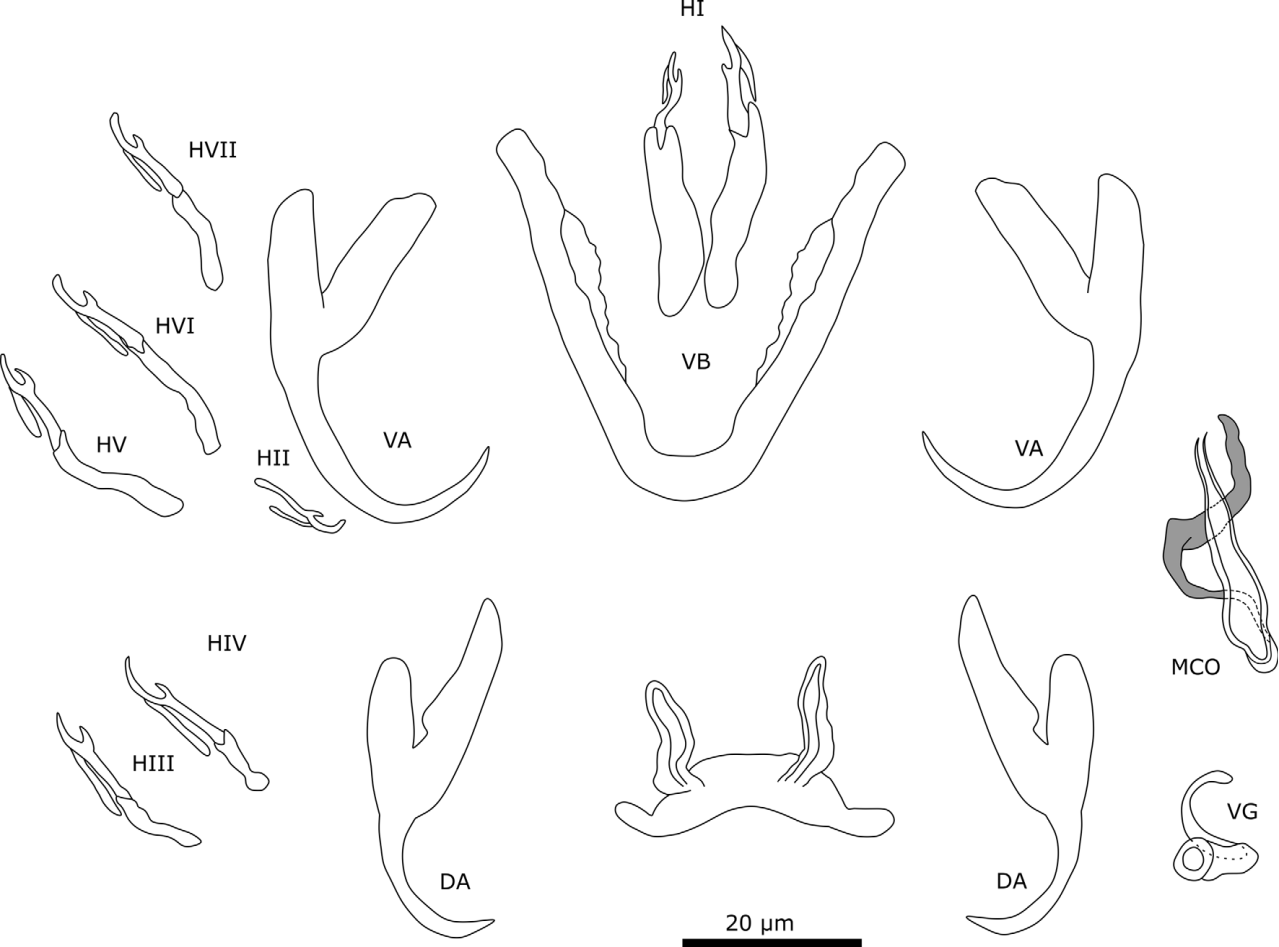
Sclerotised structures of *Cichlidogyrus tshuapa* n. sp. Abbreviations: HI-HVII, hooks; VA, ventral anchor; VB, ventral transverse bar; DA, dorsal anchor; DB, dorsal transverse bar; MCO, male copulatory organ.

#### Remarks

All specimens show diagnostic features of species of *Cichlidogyrus* (see “Remarks” *C. ataikputu* n. sp.). The new species strongly resemble *Cichlidogyrus papernastrema* Price, Peebles & Bamford, 1969 [93] infecting *Tilapia sparrmanii* Smith, 1840 [93], *Oreochromis mweruensis* Trewavas, 1983, and *Coptodon rendalli* (Boulenger, 1897). The copulatory tube of *Cichlidogyrus tshuapa* n. sp. has a slightly broadened middle section whereas, in *C. papernastrema*, this section forms a more apparent rounded bulb. In the redescription of *C. papernastrema* by Jorissen *et al.* [42], a heel is present and the accessory piece is described as S-shaped. The curvature of the accessory piece at the distal end is almost the same as in *C. tshuapa* n. sp. However, the curvature of the accessory piece in *C. tshuapa* n. sp. is narrower than in the original description [93] and the redescription [42] of *C. papernastrema*. Furthermore, the haptor differs substantially. The outer root of the dorsal anchors is larger in *C. tshuapa* n. sp. (on average 13.3 μm compared to 7 μm in *C. papernastrema* [93]), as is the inner root (25.4 μm compared to 17 μm). The ventral bar of *C. papernastrema* also lacks membranous extensions unlike in *C. tshuapa*. Furthermore, *C. tshuapa* n. sp. presents a sclerotised vagina unlike *C. papernastrema*.

### *Cichlidogyrus thysochromis* Moons, Kmentová, Pariselle, Vanhove & Cruz-Laufer n. sp.

urn:lsid:zoobank.org:act:1CF17361-0C49-47FF-B76F-DEACC1FE9175

Type host: *Thysochromis ansorgii* (Boulenger, 1901).

Type locality: Aboisso, Côte d’Ivoire; 4.47, −3.2; November 1958.

Material: 1 whole-mounted specimen fixed in Hoyer’s medium.

Holotype: RMCA_VERMES_44375.

Symbiotype: RMCA_Vert_1973.005.P.4470-4476 (447).

Infection site: gills.

Etymology: The species epithet refers to the host genus *Thysochromis*.

Note: The authors of the new taxa are different from the authors of this paper: Article 50.1 and Recommendation 50A of the International Code of Zoological Nomenclature [38].

#### Description (Table 4, Fig. 9)

Two pairs of anchors. Ventral anchor with well-developed inner root and smaller outer root. Dorsal anchors present but distorted on slide. Ventral transverse bar V-shaped. Membranous attachments attached over the length of bar. Dorsal transverse bar with thick midsection and auricles. The auricles are small and slender. Most likely seven pairs of marginal hooks like all congeners, but pair 3 was not observed due to poorly preserved specimen. First pair is larger than other hooks. Rest of the hooks are about the same size. The male copulatory organ consists of copulatory tube, accessory piece, and heel. Base of copulatory tube is broad. Copulatory tube makes a 90° turn near base and narrows distally. Heel curves around base of copulatory tube and is broad. Accessory piece is shaped drop-like and surrounds copulatory tube distally. Basal portion of accessory piece was not observed. No sclerotised vagina was observed.

**Figure 9:**
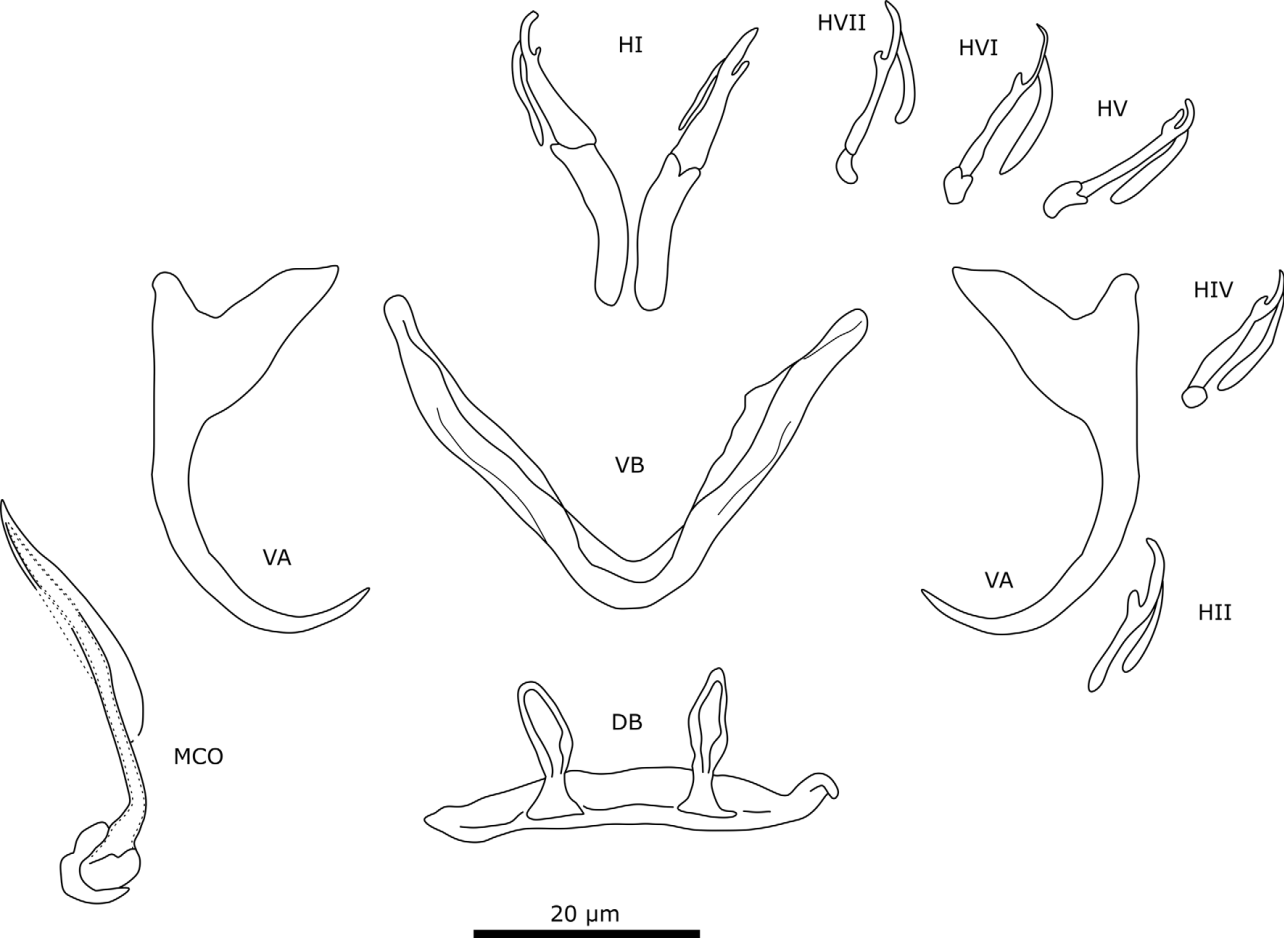
Sclerotised structures of *Cichlidogyrus thysochromis* n. sp. Abbreviations: HI-HVII, hooks; VA, ventral anchor; VB, ventral transverse bar; DA, dorsal anchor; DB, dorsal transverse bar; MCO, male copulatory organ.

#### Remarks

The only observed specimen shows diagnostic features of species of *Cichlidogyrus* (see “Remarks” *C. ataikputu* n. sp.). Although multiple fish were examined for parasites, only one specimen of this species was found. While describing a new species based on single individuals (singletons) is unusual for monogeneans, previous studies have done so with partial specimens or a few specimens when the morphology was discernible and distinct from other species [43] and the practice is widespread among other taxa, *e.g.*, arthropods [59]. Therefore, we opted to describe the present specimen as a new species. *Cichlidogyrus thysochromis* n. sp. does show some similarities, *e.g.* in the haptor, with *Cichlidogyrus polyenso* Jorissen, Pariselle & Vanhove, 2018 [43] and *Cichlidogyrus calycinus* Kusters, Jorissen, Pariselle & Vanhove, 2018 [43] both infecting *Hemichromis elongatus* (Guichenot, 1861), *Cichlidogyrus teugelsi* Pariselle & Euzet, 2004 [82] infecting *Hemichromis fasciatus* Peters, 1858, and *Cichlidogyrus reversati* Pariselle & Euzet, 2003 [77] infecting *Pelmatolapia cabrae* (Boulenger, 1899). The ventral anchors of the different species have well-developed inner roots and small outer roots. The morphology of the ventral bar of *C. polyenso* resembles that of *C*. *thysochromis* n. sp. the most, by having a membranous attachment associated with the ventral bar. The auricles of the dorsal transverse bar are of similar size as the auricles of the species mentioned above. The size of the auricles falls within the range of *C. calycinus*, *C. teugelsi*, and *C. polyenso*. Pair 1 of the marginal hooks is also similar in morphology and size in having a broad and long secondary shaft. The size of the marginal hook pair 1 of *C*. *thysochromis* n. sp. falls within the range of *C. teugelsi* and *C. reversati*. Conversely, the MCO of *C. thysochromis* n. sp. has no resemblance to the MCO of the above mentioned species. In all species, the copulatory tube is G-shaped (*C. calycinus* and *C. teugelsi*) or spiralled (*C. polyenso*), while in *C. thysochromis* n. sp., the copulatory tube is only slightly curved.

### *Cichlidogyrus* sp. “*Pelvicachromis roloffi”*

Host: *Pelvicachromis roloffi* Paperna, 1968.

Locality: Kahmranka, near Rokupr 10–15 km, route Rokupr-Kambia, Sierra Leone; 9.07, −12.93; 5/4/1969.

Material: 1 whole-mounted specimen fixed in Hoyer’s medium.

Host voucher: RMCA_Vert_P.174947-174968 (A).

Parasite material: RMCA_VERMES_44387.

#### Characterisation (Table 4, Fig. 10)

MCO consists of copulatory tube and accessory piece. Copulatory tube is straight with no heel attached to base. Distal end of copulatory tube is not observed as accessory piece is folded over its distal portion. Accessory piece shows a minor split at distal end and is attached to base of copulatory tube.

**Figure 10:**
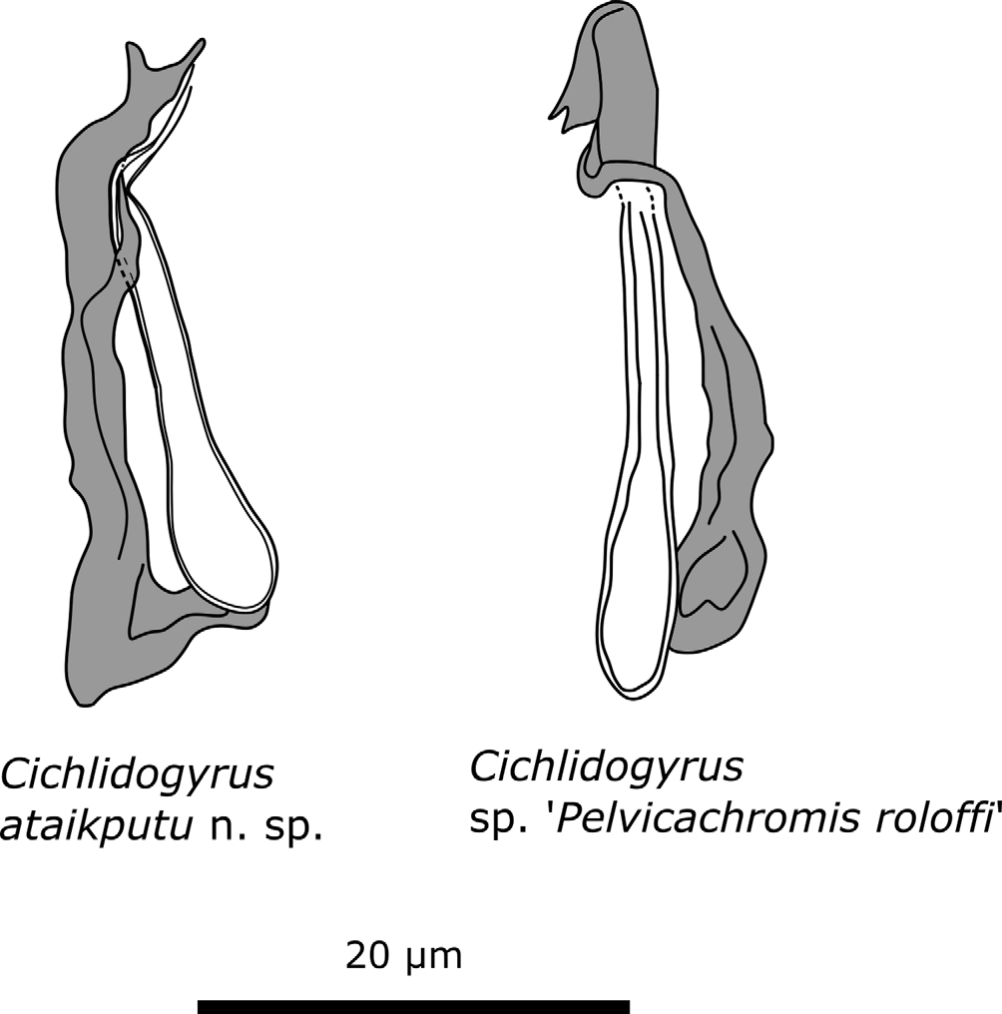
Comparison of the MCO of *Cichlidogyrus ataikputu* n. sp. and *Cichlidogyrus* sp. “*Pelvicachromis roloffi”*.

#### Remarks

Only one specimen was found on the gills of *Pelvicachromis roloffi*, but the haptor was lost during the sampling process and the MCO appeared slightly distorted, resulting from fixation on the slide. Based on this information, the specimen could not be assigned to a species or delimited from other species. The MCO resembles the MCO of *Cichlidogyrus ataikputu* n. sp. The fold over the copulatory tube is seen in *C. ataikputu* n. sp. and the split at the distal end is also present in *C. ataikputu* n. sp. The average size of the copulatory tube of *C. ataikputu* n. sp. is 25.0 μm and the average length of the accessory piece is 27.2 μm; measurements of *C*. sp. “*Pelvicachromis roloffi*” are 28.7 and 29.3 μm, respectively. These measurements fall within the range of *C. ataikputu* n. sp. However, the different attachment point of the accessory piece to the basal bulb of the copulatory tube strongly suggests that *C*. sp. “*Pelvicachromis roloffi”* is a new species. However, there is currently too little information to support a formal description.

### *Onchobdella* Paperna, 1968

#### Emended diagnosis (based on Paperna [72] and Pariselle & Euzet [83])

Body shape elongated or stout. Prohaptoral anterior lobes are poorly demarcated and head organs are present. Two pairs of eyespots that are arranged in pairs of two in front of pharynx or in one transverse row in front of pharynx. Three pairs of cephalic glands. Intestinal crura are united posteriorly. Single testis is in posterior position within intestinal loop, while single ovary is located anteriorly to testis. MCO consists of tubiform cirrus and accessory piece, consisting of two elongated bracket shaped portions. Portions are attached to each other along one or two edges and copulatory tube is often protruded between. Seminal vesicles and 2–3 prostate glands follow distal part of male genital system. Sclerotised vagina present, opening lateral. Anchors two pairs unequal in size and shape. One dorsal, large pair and one ventral small pair more hook shaped. Three transversal bars, one sturdy dorsal bar that is either slightly curved or horse-shoe shaped, and a ventral bar split in two, frequently curved bars. Each anchor is accompanied by membranous filaments, attached to shaft. Seven pairs of marginal hooks all approximately the same length with thin needle-shaped shaft with delicate posterior projecting process.

#### Remarks

Species of *Onchobdella* are reported from *Chromidotilapia guntheri* (Sauvage, 1882), *Pelmatochromis buettikofferi* (Steindachner, 1894), and species of *Hemichromis* Peters, 1857; and are mainly found in West and Central Africa. The genus was created in 1968 to include five new species (*O. aframae* Paperna, 1968, *O. krachii* Paperna, 1968, *O. pterigyalis* Paperna, 1968, *O. spirocirra* Paperna, 1968, and *O. voltensis* Paperna, 1968 [72]). Paperna considered the presence of two pairs of anchors of unequal size, an accessory piece consisting of two elongated bracket shaped portions, and having three transversal bars as the main characteristics in the first diagnosis [72]. Since Paperna’s diagnosis, six new species (*O. bopeleti* Bilong Bilong & Euzet, 1995 [3], *O. melissa* Pariselle & Euzet, 1995 [83], *O. sylverai* Pariselle & Euzet, 1995 [83], and *O. ximenae* Jorissen, Pariselle & Vanhove in Jorissen *et al.* [43]) were described, including the two new species described here. As several characteristics deviate from the original diagnosis, we provide an emended diagnosis here. In the original diagnosis, the dorsal bar was described as a frequently curved bar. The first species of *Onchobdella* that were described had either horseshoe-shaped or slightly curved dorsal bars [72]. The dorsal bar is horseshoe-shaped in *O. voltensis*, *O. spirocirra*, *O*. *pterigyalis* and *O. bopeleti* unlike the slightly curved dorsal bar of *O. aframae*, *O. krachii*, *O. melissa,* and *O. silverai*. The ventral bar is split in two. Two pairs of eyes are observed in *O. macrohamuli* n. sp., *O. yemojae* n. sp., *O. krachii*, *O. silverai* and *O. melissa* [83], unlike the single pair suggested by Paperna [72]. Lastly, 14 marginal hooks are counted in the species described after Paperna [72], while the original diagnosis mentions only 4–6 pairs of marginal hooks. Pariselle and Euzet [83] already remarked on this difference. Notably, the hooks are difficult to count correctly as the large dorsal anchors often conceal their presence.

### *Onchobdella macrohamuli* Moons, Kmentová, Pariselle, Vanhove & Cruz-Laufer n. sp.

urn:lsid:zoobank.org:act:8AA83C9E-2420-4CEC-BA39-8E229402748C

Type host: *Thysochromis ansorgii* (Boulenger, 1901) (Perciformes: Cichlidae).

Type locality: Oshika, 10 km NW of Ahoada, Nigeria; 5.12, 6.63; March 1984.

Additional locality: Attingué, Agnébi basin, Côte d’Ivoire; 5.47, -4.183 and Whedda, Ouémé river, Benin, 6.75, 2.457 on *Thysochromis ansorgii.*

Material: 5 whole-mounted specimens fixed in Hoyer’s medium.

Holotype: RMCA_VERMES_44371.

Paratypes: RMCA_VERMES_44374, RMCA_VERMES_44383, HU 846, SAMC-A095105.

Symbiotype: RMCA_Vert_1984.022.P.0012-0014 (A).

Symbioparatype: RMCA_Vert_1973.005.P.4470-4476 (447); RMCA_Vert_1973.005.P.4478-4503 (502).

Infection site: gills.

Etymology: The species epithet is a combination of the Greek word “*macro*” (= long) and the Latin word “*hamulus*” (= hook-shaped carpal bone). The combination is used to describe the exceptionally large dorsal anchors for species of *Onchobdella*.

Note: The authors of the new taxa are different from the authors of this paper: Article 50.1 and Recommendation 50A of the International Code of Zoological Nomenclature [38].

#### Description (Table 5, Fig. 11)

Dorsal anchors are very large. Outer root of large anchor is reduced while inner root is well-developed. Outer roots of small (ventral) anchors are more developed, but still smaller than inner roots. Dorsal bar stout and straight, slightly curved at both ends. Ventral bars slightly curved. Seven pairs of hooks all approximately the same length and thin, needle-like. Male copulatory organ consists of copulatory tube and accessory piece. Copulatory tube strongly curved with broad, bean-shaped base. At accessory piece, copulatory tube is almost straight. Base of accessory piece connects to base of copulatory tube. Accessory piece consists of two portions that connect to each other at base. Tips of two portions are slightly curved at distal end and do not connect with each other. No sclerotised vagina observed.

**Figure 11:**
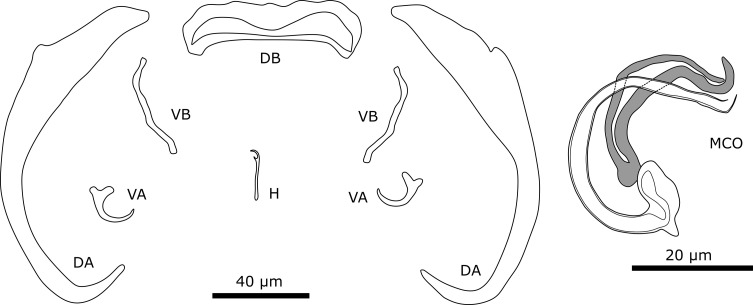
Sclerotised structures of *Onchobdella macrohamuli* n. sp. Abbreviations: H, marginal hook; VA, ventral anchor; VB, ventral transverse bar; DA, dorsal anchor; DB, dorsal transverse bar; MCO, male copulatory organ.

**Table 5:** Morphometrics of species of *Onchobdella* infecting chromidotilapiines cichlids. min–max, minimum and maximum value; SD, standard deviation; *n*, sample size.

Measure	*Onchobdella krachii*				*Onchobdella macrohamuli* n. sp.				*Ochobdella yemojae* n. sp.			
	mean	min–max	SD	*n*	mean	min–max	SD	*n*	mean	min–max	SD	*n*
DAa	37.6	29.1–46.8	4.6	51	120.0	95.6–131.7	14.1	5	39.4	33.2–45	3.6	20
DAb	26.7	19.4–31.1	2.6	49	102.5	92–107.2	6.2	5	29.5	24.3–34.6	3	19
DAc	2.8	1.1–5.1	0.9	47	4.3	2.3–7	1.8	5	2.2	1–5.5	1.4	18
DAd	15.9	10.2–22.2	2.8	51	28.8	14.6–45.4	13.5	5	13.7	11.3–17	1.7	19
DAe	16.3	10.8–19.8	2.5	52	28.1	24.9–31.2	2.6	5	20	15.7–25.2	2.4	21
DBw	5.5	3.2–7.7	1.1	57	17.8	14.2–21.6	3.4	5	5	2.9–9.7	1.5	22
DBx	41.3	23.9–54.6	7.3	56	70.4	60.1–79.1	7.3	5	38.5	31.4–49.8	5.5	22
VAa	13.8	11–17.3	1.1	53	15.5	14.5–16.4	0.9	5	8	6.6–9.7	1	21
VAb	11.8	9.5–15.1	0.8	53	16.1	14.6–18.6	1.7	5	7.9	6.6–9.5	0.9	21
VAc	2.3	1.2–4	0.6	53	3	2.5–4.1	0.7	5	1.6	0.8–2.3	0.4	20
VAd	6.5	5.2–8.3	0.8	53	5.3	4–8.2	1.6	5	4.1	3.3–5.4	0.5	21
VAe	6.8	5.2–8.8	0.8	53	7.1	4.5–9.8	2.3	5	4.2	2.6–5.5	0.8	20
VBw	3.1	1.9–4.8	0.6	52	2.7	1.9–3.4	1.1	2	1.5	1.3–1.8	0.2	11
VBx	30.5	19.2–41.6	4.6	52	42.5	40.4–44.6	3	2	20.2	16.3–22.4	1.8	9
H	14.3	11.9–16.3	1.2	19	16.5	15.7–16.9	0.5	4	13.4	10.5–15.5	1.9	5
Pe	53.3	33.1–99.8	11.8	66	65.4	–	1	2	36.6	28.8–45.2	4.7	17
AP	21.1	14.1–41.6	3.8	63	32.3	27.7–36.8	6.4	2	30.3	21–44.8	6.8	17

#### Remarks

The specimens show typical features of members of *Onchobdella*, *i.e.*,: (i) two pairs of anchors unequal in size, one larger and one smaller pair; (ii) two ventral bars, club shaped; (iii) one dorsal bar, curved solid; and (iv) hooks seven pairs, needle-shaped [81]. *Onchobdella macrohamuli* n. sp. shows similarities with *Onchobdella krachii* Paperna, 1968 infecting *Chromidotilapia guntheri* (Sauvage, 1882) and *Chromidotilapia linkei* Staeck, 1980. The species has exceptionally large dorsal hooks (DAa on average: 120.0 μm), three times the length of those of *O. yemojae* n. sp. (Table 5), which is found on the same host species (see description below). Other components of the haptor are in the size range that is known from previously described species of *Onchobdella*. The dorsal bars of *O. macrohamuli* n. sp. are different from those of *O. krachii*, whose dorsal bars are broad and straight with curved edges. The morphology of the dorsal anchor is different from *O. krachii*. The outer root is almost not developed in *O. macrohamuli* n. sp. unlike in *O. krachii*. Ventral bars of *O. macrohamuli* n. sp. are more curved than those of *O. krachii*. The two portions of the accessory piece are, however, not connected at the distal end in *O. macrohamuli* n. sp., unlike in the other two species. In *O. krachii*, the base of the copulatory tube is round.

### *Onchobdella yemojae* Moons, Kmentová, Pariselle, Vanhove & Cruz-Laufer n. sp.

urn:lsid:zoobank.org:act:5C4A6CFD-B268-4BAA-8D5F-79E4A74546BD

Type-host*: Thysochromis ansorgii* (Boulenger, 1901) (Perciformes: Cichlidae).

Type locality: Whedda, Ouémé river, Benin; 6.75, 2.47; 14/10/1966.

Additional locality: Attingué, basin Agnébi, Côte d’Ivoire; 5.47, −4.183 and Oshika, 10 km NW of Ahoada, Nigeria; 5.117, 6.633 on *Thysochromis ansorgii.*

Material: 29 whole-mounted specimens fixed in Hoyer’s medium.

Holotype: RMCA_VERMES_44381.

Paratypes: RMCA_VERMES_44370, RMCA_VERMES_44372–44373, RMCA_VERMES_44376–44380, RMCA_VERMES_44382, RMCA_VERMES_44384, HU 843–845, MZH http://id.luomus.fi/KN.37258–http://id.luomus.fi/KN.37260, SAMC-A095106–A095108, MNHN HEL1906–1907.

Symbiotype: RMCA_Vert_1973.005.P.4478-4503 (447).

Symbioparatype: RMCA_Vert_1973.005.P.4470-4476 (500, 502, 503); RMCA_Vert_1984.022.P.0012-0014 (A).

Infection site: gills.

Etymology: The species epithet is based on the name of the water spirit Yemoja from the Yoruba religion. This religion originates in the countries where *O. yemojae* n. sp. is found but has since then spread to other parts of the world as a result of the Yoruba diaspora.

Note: The authors of the new taxa are different from the authors of this paper: Article 50.1 and Recommendation 50A of the International Code of Zoological Nomenclature [38].

#### Description (Table 5, Fig. 12)

Two pairs of anchors. Dorsal and ventral anchors have well-developed inner root and reduced outer root. Ventral bars are thin and slightly curved, middle portion is straight with both ends being curved. Dorsal bar curves in middle and ends are straight. Seven pairs of thin marginal hooks of similar size. MCO consists of copulatory tube and accessory piece. Copulatory tube has bean-shaped basal bulb and narrows towards distal end. Accessory piece consists of two plates that connect at proximal end, where it also attaches with base of copulatory tube.

**Figure 12:**
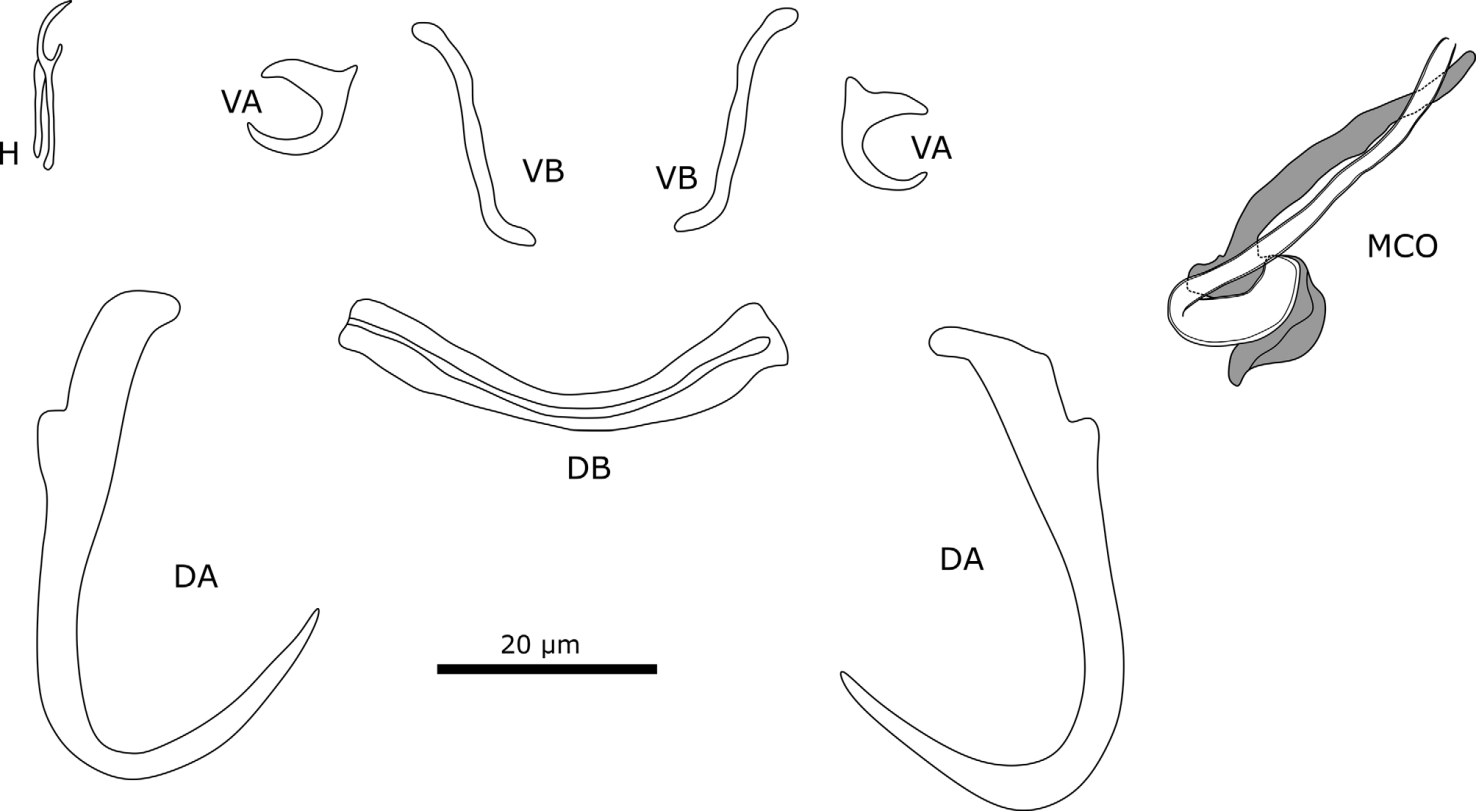
Sclerotised structures of *Onchobdella yemojae* n. sp. Abbreviations: H, marginal hook; VA, ventral anchor; VB, ventral transverse bar; DA, dorsal anchor; DB, dorsal transverse bar; MCO, male copulatory organ.

#### Remarks

All specimens show diagnostic features of species of *Onchobdella* (see Remarks on *O. macrohamuli* n. sp.). *Onchobdella yemojae* n. sp. resembles *Onchobdella melissa* Pariselle & Euzet, 1995 infecting *Pelmatochromis buettikoferi* (Steindachner, 1894). The morphology of the haptor of both species is similar. For instance, the dorsal bar is curved slightly with straight ends. However, *O*. *yemojae* n. sp. shows a ridge along the dorsal bar similar to *O. krachii*, whereas *O. melissa* lacks this ridge. In *O. krachii*, the bar has bent instead of straight ends. The morphology of the anchors is also similar as both *O. melissa* and *O. yemojae* n. sp. have reduced outer roots and well-developed inner roots. Furthermore, the ventral bars present rounded, slightly bent ends in both *O. krachii* and *O. yemojae* n. sp. The accessory piece of *O*. *yemojae* n. sp. is connected to the base of the copulatory tube. In *O. melissa*, the accessory piece is not connected to the base of the copulatory tube and the plates of the accessory piece are of different lengths. The copulatory tube of *O. melissa* is G-shaped, while the copulatory tube of *O. yemojae* n. sp. is J-shaped. The copulatory tube in *O. melissa* is also longer (69 μm ± 2.9 μm [83]) than in *O. yemojae* n. sp. (36.6 μm ± 4.7 μm). The typical structure, two portions connected at the base (accessory piece), is difficult to observe in *O*. *yemojae* n. sp. as only one of the plates is visible in most specimens.

### *Onchobdella krachii* Paperna, 1968

Type locality: Kpandu and Kete Krachi, Volta Lake, Ghana.

Present localities: New Calabar river, Akpor, Nigeria; 4.87, 6.9; 01/12/1990 on *Chromidotilapia guntheri* (Sauvage, 1882); road Yabassi–Yingui, Cameroon; 07/11/1990 on *Chromidotilapia linkei.*

Material: 82 whole-mounted specimens fixed in Hoyer’s medium.

Parasite material: RMCA_VERMES_44388–44390, RMCA_VERMES_44392–44393, RMCA_VERMES_44397–44398, RMCA_VERMES_44400–44401, RMCA_VERMES_44403–44406, RMCA_VERMES_44408–44410, RMCA_VERMES_44416–44419, RMCA_VERMES_44426–44427, RMCA_VERMES_44436–44441, RMCA_VERMES_44443–44444, RMCA_VERMES_44446, RMCA_VERMES_44448–44452, RMCA_VERMES_44467–44490, HU XIX.2.16a–XIX.2.16c, HU XIX.2.18-XIX.2.19b, MZH http://id.luomus.fi/KN.37263–http://id.luomus.fi/KN.37265, MZH http://id.luomus.fi/KN.37272–http://id.luomus.fi/KN.37273, SAMC-A095111–A095115, MNHN HEL1908–1910.

Host vouchers: RMCA_Vert_1991.010.P.0542-0582 (576, 577, 578, 580, 581, 582); RMCA_Vert_1992.144.P.0250-0261 (A, B, C).

Infection site: gills.

#### Redescription (Table 5, Fig. 13)

Two pairs of anchors. Dorsal anchors have well-developed inner roots and reduced outer roots. Ventral anchors have well-developed inner roots and small outer roots. Dorsal bar is slightly curved at both ends of bar. Ventral bars are also slightly curved with small indentation at one end. Seven pairs of marginal hooks present and all approximately of the same size, thin and needle-shaped. MCO consists of copulatory tube and accessory piece. Copulatory tube draws a spiral in the shape of the letter G and has oval-shaped base. Accessory piece is attached to base of copulatory tube and consists of two plates that connect at proximal end. At distal end, a structure is connected to one of the plates running in parallel with copulatory tube.

**Figure 13:**
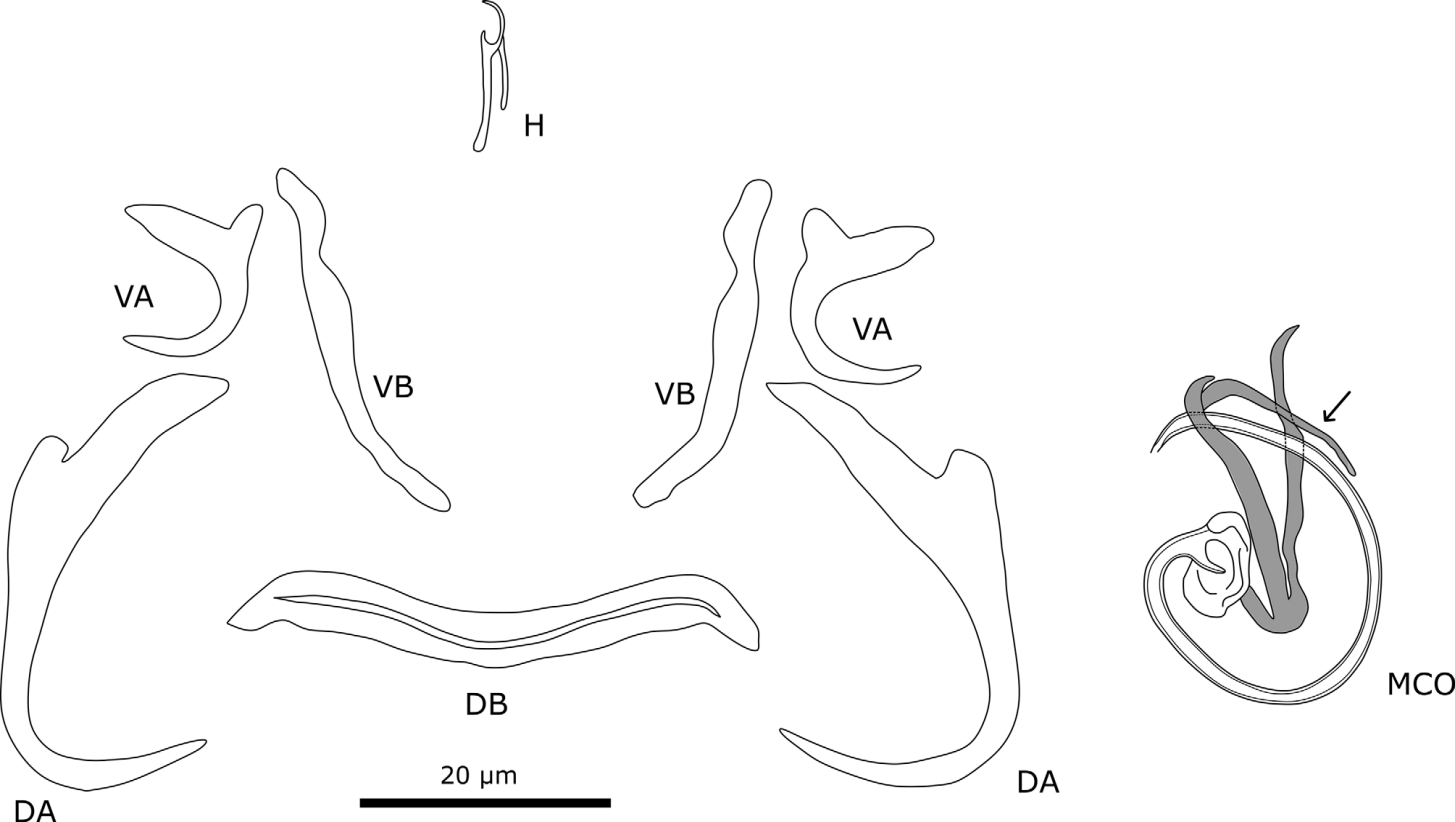
Sclerotised structures of *Onchobdella krachii*. Abbreviations: H, marginal hook; VA, ventral anchor; VB, ventral transverse bar; DA, dorsal anchor; DB, dorsal transverse bar; MCO, male copulatory organ. Arrow indicates additional structure of the MCO that was missing from previous characterizations.

#### Remarks

All specimens show diagnostic features of species of *Onchobdella* (see Remarks on *O. macrohamuli* n. sp.). The original description by Paperna [72] was based on two specimens. Here, we studied 82 specimens and found additional characteristics. The small indentations in the ventral bars were not reported before. Furthermore, the accessory piece of the MCO differs in small characteristics from the original description. The two plates do not connect with the copulatory tube unlike originally reported. Also, an additional structure has been discovered that is connected to one of the plates (Fig. 13).

### Morphometrics

**Figure 14:**
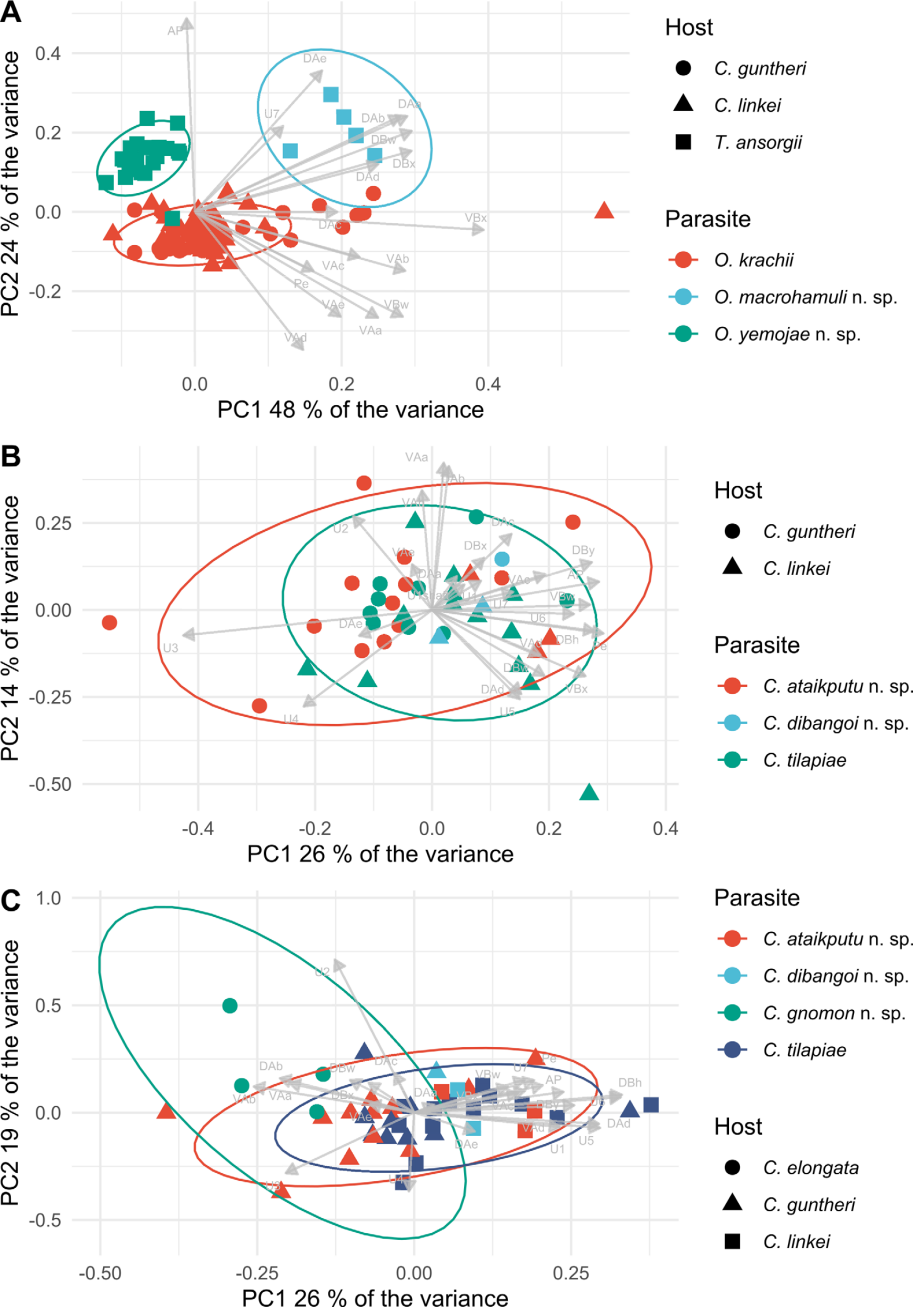
Principal component analyses of monogenean flatworms infecting chromidotilapiine cichlids. A, Species of *Onchobdella* showing three distinct clusters that are mostly congruent with the species identities assigned in this study. B, Several species of *Cichlidogyrus* strongly resemble *C. tilapiae*, but at least two of them form distinct species. C, Species of *Cichlidogyrus* infecting chromidotilapiine cichlids form distinct clusters in the PCA, albeit with some overlap.

We produced three plots for the principal component analysis (PCA) to test whether the qualitative distinctions between the species infecting chromidotilapiine cichlids translate into morphometric differences between their attachment and reproductive organs. We conducted one PCA including all species of *Onchobdella* sampled here, one with species similar to *Cichlidogyrus tilapiae* including *C. tilapiae* ex *Chromidotilapia* spp., *Cichlidogyrus dibangoi* n. sp*.*, and *C. ataikputu* n. sp*.*, and one with all species of *Cichlidogyrus* sampled in the present study (Fig. 14).

Specimens belonging to *Onchobdella* (first two principal components together explaining overall 72% of the variation) confirmed the differentiation of the three chromidotilapiines-infecting species. We found two well-separated clusters, with *Onchobdella macrohamuli* n. sp. differing substantially from the other species in the size of the dorsal anchor and the dorsal bar. The clusters produced by specimens of *Onchobdella krachii* and *O. yemojae* n. sp. overlap slightly, but with a visible difference in the measurements of the dorsal anchor (Fig. 14A), one of the characters we highlighted in the description of *O. macrohamuli* n. sp.

**Figure 15:**
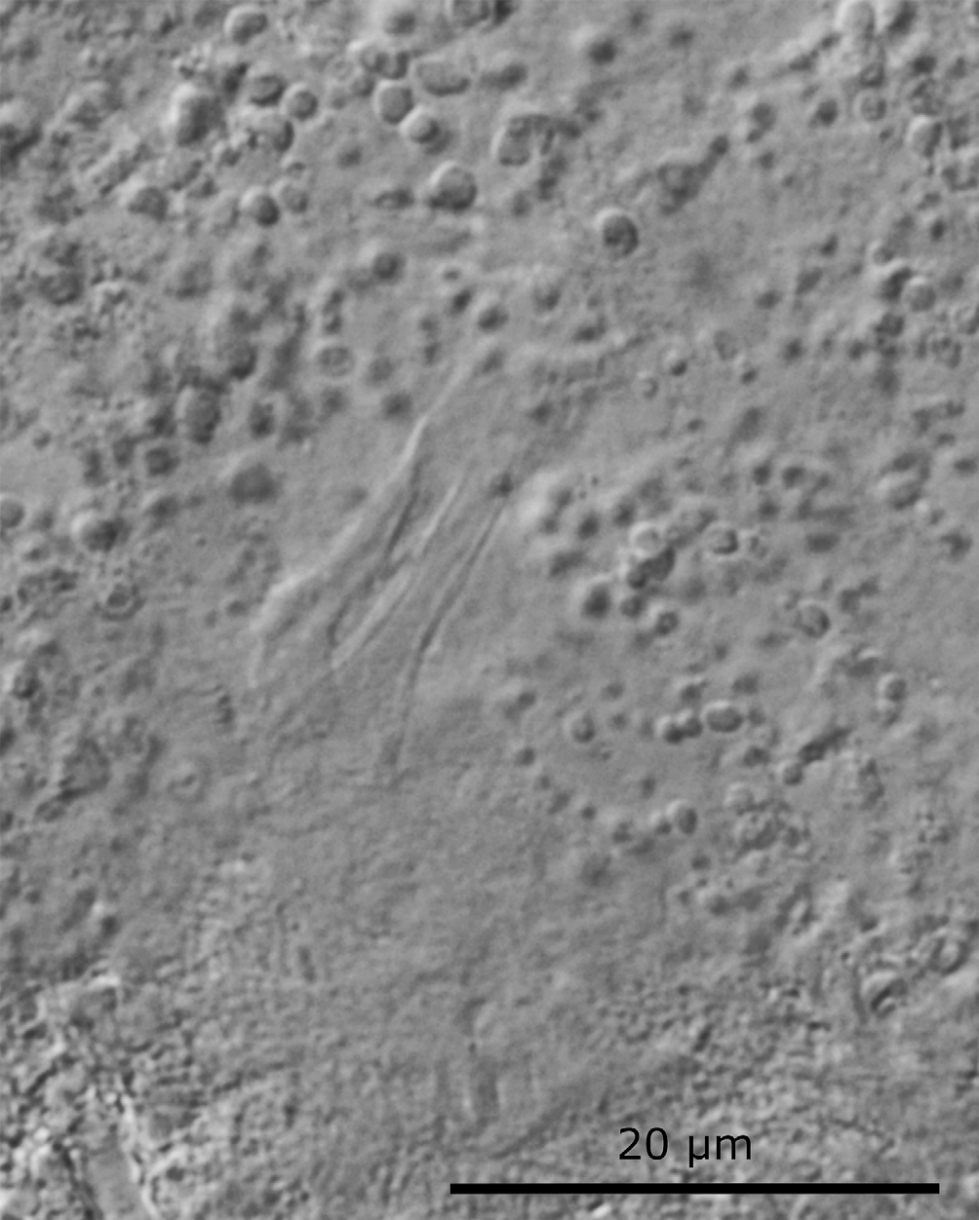
Male copulatory organ (MCO) of a specimen of *Cichlidogyrus tilapiae* infecting *Chromidotilapia guntheri* from Lake Barombi-Kotto.

*Cichlidogyrus tilapiae* on one side and the *C. ataikputu* n. sp. and *C. dibangoi* n. sp. on the other side present no apparent clustering (first two PCs explaining 40% of the variation) (Fig. 14B). The pattern indicates that *C. tilapiae* is indistinguishable from the other two species based purely on morphometric characters, highlighting the importance of the qualitative characters used in this study. Specimens of *C. tilapiae* found in this study (Fig. 15) were not morphologically distinguishable from specimens from previous studies (as redescribed in [117]) although the measurements differed considerably. Despite the difference in size, we still treat these specimens as belonging to *C. tilapiae* because of a lack of qualitative morphological differences. Finally, when comparing species of *Cichlidogyrus* infecting species of *Chromidotilapia* (first two PCs explaining 47% of the variation), we did not detect any apparent clusters (Fig. 14C).

### Phylogenetic position

**Figure 16:**
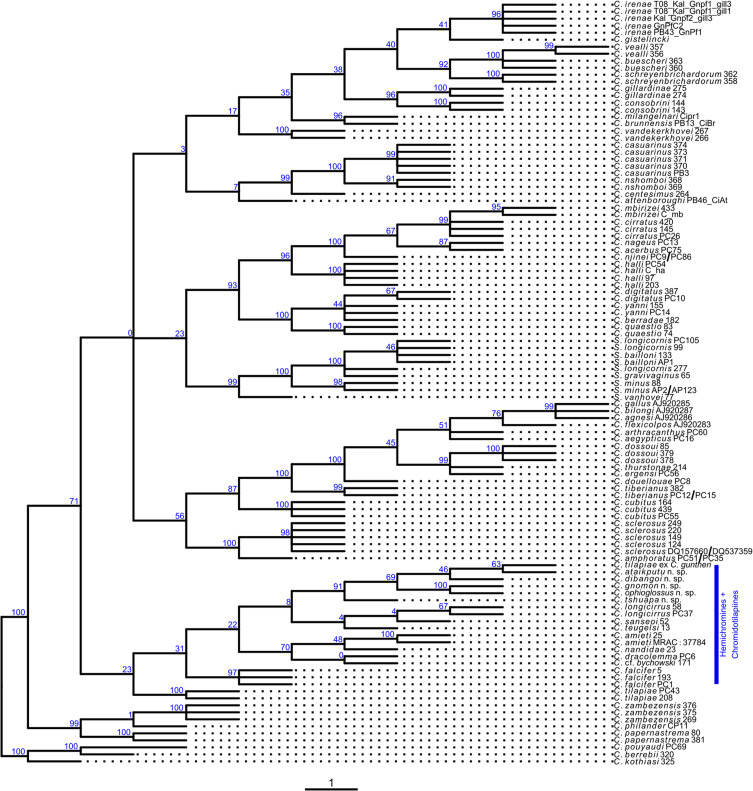
Phylogram of species of *Cichlidogyrus* inferred from morphological characters under maximum parsimony and using the molecular tree published by Cruz-Laufer *et al.* [12] as a constraint (specimen IDs refer to samples from Cruz-Laufer et al. [12]). Species of *Cichlidogyrus* infecting chromidotilapiines cichlids appear nested inside a species group infecting mostly hemichromine cichlids (highlighted in blue), a host tribe with a shared distribution in Central and West Africa.

According to the parsimony analysis, the species of *Cichlidogyrus* infecting chromidotilapiine cichlids form a well-supported monophyletic group (GC = 95), including specimens identified as *Cichlidogyrus tilapiae* (Fig. 16). This group appears firmly nested inside a well-supported (GC = 31) clade of Western African species known to infect mostly hemichromine cichlids, see “*Hemi*” group *sensu* [12]. Measurements of *C. thysochromis* n. sp. were not included in this analysis due to large amount of missing data.

The machine learning algorithms predicted clade affiliation of species of *Cichlidogyrus* with known clade affiliation with varying degrees of accuracy. Random Forest showed a moderate agreement (*κ* = 0.51) for species with known phylogenetic position after parameter optimisation (mtry = 9) followed by artificial neural networks (*κ* = 0.39; size = 20, decay = 1E-04) and support vector machines (*κ* = 0.24; *C* = 3.05E–05, *σ* = 8192) with only fair agreements. However, new species of *Cichlidogyrus* described here were placed in different groups (*Bulb*, *EAR*, *Oreo1, Tilapiae*, *Tylo*, see [12]) but never close to species infecting hemichromine cichlids, as suggested by the parsimony analysis.

## Discussion

Chromidotilapiini is one of the most species-rich tribes of cichlid fishes on the African continent, rivalled only by the hyperdiverse lineages of the Eastern African radiations and tilapias from Oreochromini [108, 111]. Despite this diversity, their relationship with other organisms in their environment remains poorly understood, especially concerning parasitic diseases. Forty years have passed since the last major parasitological studies on chromidotilapiines [74, 75] and almost 30 years since the most recent infection was reported in the literature [76]. Here, the parasite fauna of chromidotilapiines across West and Central Africa is investigated for the first time. Furthermore, this study is the first to infer the phylogenetic position of new monogenean species without molecular data using a phylogenetic analysis restricted by a baseline molecular phylogeny. Through examining the gills of specimens stored in natural history collections, ten species of dactylogyrid monogeneans were reported, of which eight are new to science, six belonging to *Cichlidogyrus* and two to *Onchobdella*.

### Monogenean evolution in Western Africa: allopatric speciation

The evolutionary history of monogenean parasites infecting cichlid fishes in West and Central Africa has been largely shaped by geographical constraints. Specifically, among the lineages of *Cichlidogyrus* and *Onchobdella* infecting chromidotilapiine, hemichromine, and pelmatochromine cichlids, we find strong indicators for allopatric speciation processes. Chromidotilapiini, Hemichromini, and Pelmatochromini are tribes of cichlids whose members occur across Central and West Africa [4, 55, 111]. Our study reveals that their monogenean parasites belonging to *Cichlidogyrus* and *Onchobdella* are each other’s closest relatives. Therefore, the parasite lineages have likely diverged from their relatives infecting other cichlid fishes due to the geographical isolation of the host lineages. While this pattern might also be explained by the fact that the host lineages diverged early from the haplotilapiine lineages [110, 111], chromidotilapiines, hemichromines, and pelmatochromines share no common ancestor. Therefore, co-speciation of host and parasite lineages can be excluded. The close relationship of the parasite lineages is not the result of shared ancestry of the host species. Instead, the monogenean fauna is indicative of the shared environment where chromidotilapiines, hemichromines, and pelmatochromines occur. The incongruence of parasite and host phylogenies stands in contrast with species of *Cichlidogyrus* infecting hosts from the East African radiations, where both hosts and parasites form well-supported monophyletic clades [12, 97]. Allopatric patterns in the evolution of West and Central African monogeneans come as no surprise as this extensive region offers a multitude of ecological barriers and encompasses many isolated habitats, such as river basins [54, 56, 120, 128], lakes [62, 101], and rapids [112], all of which support high numbers of endemic fishes.

Despite their similar distribution patterns, the lineage of *Cichlidogyrus* infecting chromidotilapiines and hemichromines, and the genus *Onchobdella* (the representatives of which additionally infect pelmatochromines) are distinct groups among Dactylogyridae. Species of *Onchobdella* were first described in 1968 from species of *Hemichromis* and *Chromidotilapia guntheri* [72]. In the following decades, several species were added that were found to infect other hemichromine [3, 42] and pelmatochromine cichlids [83]. Species of *Onchobdella* have not been reported from any other host tribes, despite several parasitological studies on oreochromine and coptodonine cichlids in West and Central Africa [43, 78, 80, 81, 84, 85]. Species of *Onchobdella* are also absent from all Eastern African cichlids [26, 98, 99] and tilapia-like cichlids across Africa [42], to our current knowledge. In contrast, species of *Cichlidogyrus* occur across Africa and the Levant [11]. Only one lineage of *Cichlidogyrus* infects the three West and Central Africa host tribes discussed here as inferred from our phylogenetic analysis (Fig. 16) wherein *Cichlidogyrus ataikputu* n. sp., *C. dibangoi* n. sp., *C. ophioglossus* n. sp., and *C. gnomon* n. sp., as well as *C. tilapiae* ex *Chromidotilapia* spp. appear as closely related to their congeners infecting hemichromine cichlids based on morphological characters of the sclerotised attachment and reproductive organs (Fig. 16). These geographical distribution patterns of species of *Cichlidogyrus* and *Onchobdella* infecting hemichromines have previously been remarked upon [43]. However, the authors of these studies remained cautious about drawing any wider conclusions because of the substantial gaps concerning monogenean biodiversity in Western Africa. Among other monogeneans, allopatric patterns are well-documented, such as those in European members of *Gyrodactylus* [35, 61]. Yet the substantial lack of knowledge in terms of species numbers and distribution patterns severely limits the possibilities for similar studies on monogenean lineages from the tropics.

Despite the results of the phylogenetic analysis, no qualitative morphological evidence of characters shared by chromidotilapiines-infecting species of *Cichlidogyrus* and their hemichromine-infecting congeners (*Hemi* clade, see [12]) was found. For example, the looped or spiralled copulatory tube, typical for the members of the *Hemi* clade, represents a feature that is absent from the chromotilapiine-infecting species. Such discrepancies may also explain the ambiguous nature of the results obtained through the machine learning analysis across all algorithms employed in this study. Furthermore, few morphological similarities were revealed between chromidotilapiines-infecting species. *Cichlidogyrus ophioglossus* n. sp. and *C. gnomon* n. sp. share a distal plate that is associated with the accessory piece of the MCO. *Cichlidogyrus ataikputu* n. sp.*, C. dibangoi* n. sp., and *C.* sp. *“Pelvicachromis roloffi”* have a simple *bauplan* (a mostly straight accessory piece and copulatory tube), reminiscent of the “tilapia” parasite *C. tilapiae*. Finally, the copulatory organ morphology of *C. tshuapa* n. sp. is reminiscent of the spiralling accessory piece of *C. papernastrema*, perhaps suggestive of a link to a group of southern African species including *C. philander* Douëllou, 1993 and *C. zambezensis* Douëllou, 1993 [12]. However, previous studies indicate that some morphological similarities in the attachment and reproductive organs of dactylogyrid monogenean may in fact be a result of convergent evolution, such as the marginal hook length in species of *Cichlidogyrus* [12], the retention of ancestral features, *e.g.*, the characters of the polyphyletic genera *Demidospermus* Suriano, 1983, *Haliotrema* Johnston & Tiegs, 1922*,* and *Ancyrocephalus* Creplin, 1839 [18, 48, 49], or host switching, *e.g.*, hook lengths in *Cichlidogyrus amieti* Birgi & Euzet, 1983 [66]. Checking for such potential contradictions between morphological patterns and subsequent molecular-phylogenetic results, once genetic data are available, is an important research target for future studies. In particular, congruence analyses [49] or phylogenetic comparative methods [12, 46, 105] as applied by previous studies might provide further insight into the evolutionary history of this lineage of *Cichlidogyrus*.

### One host, several parasites: intra-host speciation and host switching

Alongside the discussed allopatric mechanisms, several instances of host sharing of species of *Cichlidogyrus* and *Onchobdella* were identified in the current study. Host sharing can result from intra-host speciation and host switching. Recent publications indicate that parasites undergo cycles of niche isolation (*e.g.*, intra-host speciation) and expansion of host repertoires (*e.g.*, host switches) [8]; this fluctuation is also considered a likely occurrence in monogenean flatworms [6, 10]. However, identifying patterns for intra-host speciation or host switching in the absence of DNA sequence data poses a major challenge. In the present case, the differentiation in the attachment organs might signal an adaptation to specific microhabitats similar to reports from Europe on species of *Dactylogyrus* and *Lamellodiscus* Johnston & Tiegs, 1922 [88, 116]. For instance, *Onchobdella macrohamuli* n. sp. has a much larger set of dorsal anchors than the co-infecting *O. yemojae* n. sp. or any other species of *Onchobdella*. This difference might suggest an adaptation to a different gill microhabitat, where larger sclerites with more leverage (see [104]) are required, but this feature may equally be an ancestral character from a separate lineage of *Onchobdella*. No species with a similar morphology have been found to date and currently DNA sequences for only two species of *Onchobdella* are available [65]. This lack of data means that the phylogenetic relationships of species of *Onchobdella* remain currently in obscurity. Similar questions to those for *Onchobdella* also arise for co-occurrences of species of *Cichlidogyrus* on chromidotilapiines, although, in this study, the species found on chromidotilapiines appear to form a monophyletic group (Fig. 16). Co-infections of members of *Cichlidogyrus* with members of *Onchobdella* are similar to co-infections of these same groups reported for hemichromine and pelmatochromine cichlids [43, 83]. *Onchobdella* and *Cichlidogyrus* form part of Dactylogyrinae *sensu* Kmentová & Cruz-Laufer *et al.* [49], a subfamily of Dactylogyridae, but are otherwise unrelated. Niche specialisation may represent a strategy to avoid competition and to facilitate co-infections of closely related monogeneans [106, 107, 115] resulting in microhabitat preferences [25]. For instance, host sharing between species of *Cichlidogyrus* and *Onchobdella* may be indicative of distinct ecological niches on the hosts’ gills, thereby enabling the co-existence of the two lineages. A detailed analysis of the gill microhabitats such as in [25, 116] should be implemented to provide more insight into the niche habitats of these monogenean species.

### Taxonomic status of *Chromidotilapia guntheri loennbergi*

The crater lakes in Cameroon have long been of interest to evolutionary biologists as they represent not only the location of one of the most prominent examples of sympatric speciation, but are also home to many endemic species [5, 62, 69, 101]. Species of *Coptodon* and *Sarotherodon* have formed four species flocks in a total of some 25 species in lakes Bermin, Ejagham, and Barombi Mbo [62]. In the present study, the gills of specimens of *C. guntheri loennbergi* (Trewavas, 1962), which is endemic to Lake Barombi Kotto were also screened. Unlike the other lakes, Barombi Kotto supports only few species, of which *Coptodon kottae* (Lönnberg, 1904) and *C. guntheri loennbergi* are the only endemic examples [5, 122], although the status of the latter – *C. guntheri loennbergi –* has been contested due to the absence of apparent morphological delimiters [55]. Monogeneans have shorter generation time than their hosts, making them a more efficient subject of study when differentiating host population structure (*magnifying glass effect*, see [21]). Yet *Cichlidogyrus ataikputu* n. sp. and *C. dibangoi* n. sp. occur both in the lake and the surrounding river systems suggesting a lack of differentiation. Therefore, our observations provide additional evidence that *Chromidotilapia guntheri loennbergi* should be considered a synonym of *C. guntheri*.

### Opportunities and limitations of natural history collections and morphological data

The present manuscript represents the most extensive study on monogeneans infecting West and Central African fishes based solely on historic host collections to date. However, the two methods employed to identify the phylogenetic position of the newly described species of *Cichlidogyrus* (parsimony and machine learning analyses) showed varying levels of success. While the parsimony analysis provided results indicative of the well-supported hypothesis that chromidotilapiine-infecting species are closely related to their congeners infecting hemichromine cichlids, the machine learning approach that showed promise in a recent publication [12], failed to provide any conclusive results despite the use of multiple algorithms and additional morphological characters. For the time being, we recommend that the use of these algorithms be reserved as a means to quantify the predictive power of characters, rather than serving to approximate phylogenetic positions. However, the use of geomorphometrics (*e.g.*, [50, 96, 103]) might increase the systematic informativeness extracted from the morphology of the sclerotised attachment and reproductive organs.

The phenetic approach also has its limitations in discerning potential species complexes. For instance, *Cichlidogyrus ophioglossus* n. sp. presents a string-like structure in the MCO with considerable structural variation (Fig. 4). Furthermore, species belonging to the *Cichlidogyrus tilapiae* complex [92] are characterised by a relatively simple *bauplan* of its MCO with a straight copulatory tube and accessory piece with no remarkable protuberances other than a slight distal hook. Not only do *C. ataikputu* n. sp. and *C. dibangoi* n. sp. strongly resemble *C. tilapiae,* but several specimens were found (Fig. 15) that are morphologically indistinguishable from *C. tilapiae*, despite our parsimony analysis placing these specimens in a separate lineage together with the other species of *Cichlidogyrus* described herein (*C. tilapiae* ex *Chromidotilapia* spp., see Fig. 16). This result is also confirmed by the PCA (Fig. 14), where measurements of *C. tilapiae* reported from other hosts in previous publications [12, 100] form a cluster distinct from the specimens reported here. However, no qualitative characters were found that delineate the specimens infecting chromidotilapiine cichlids as a separate species. The relationships in these (potential) species complexes may be resolved only through detailed morphological and molecular studies of the target taxon.

As the biodiversity of metazoan parasites remains vastly underexplored, the present study clearly demonstrates that collection-based studies of ectoparasites are an effective tool for describing the parasite fauna of rare hosts, despite the absence of high-quality DNA samples. Recent studies also highlight the fact that these collections provide windows into the past in terms of human-induced changes of host-parasite communities [41, 129]. Morphological and collection-based studies of these organisms, therefore, unequivocally remain an essential part of taxonomic exploration.

## Data Availability

Type material was deposited in the invertebrate collection of the Royal Museum for Central Africa (Tervuren, Belgium) (RMCA) (RMCA_VERMES_44366–44602), the collection of the Research Group Zoology: Biodiversity and Toxicology of Hasselt University (Diepenbeek, Belgium) (HU 842–853), the Finnish Museum of Natural History (Helsinki, Finland) (MZH) (MZH http://id.luomus.fi/KN.37258–http://id.luomus.fi/KN.37274), the Iziko South African Museum (SAMC) (Cape Town, South Africa) (SAMC-A095104-A095122), and the Muséum National d’Histoire Naturelle (Paris, France) (MNHN) (MNHN HEL1906–1922). The morphological data that support the findings of this study are openly available in MorphoBank at www.morphobank.org, at https://doi.org/10.7934/P4626. Phylogenetic trees and data matrices for the analysis in TNT are included as additional data in MorphoBank.

## References

[1] Benovics M, Desdevises Y, Vukić J, Šanda R, Šimková A. 2018. The phylogenetic relationships and species richness of host-specific *Dactylogyrus* parasites shaped by the biogeography of Balkan cyprinids. Scientific Reports, 8, 13006. 30158640 10.1038/s41598-018-31382-w PMC6115452

[2] Bilong Bilong C, Birgi E, Euzet L. 1994. *Urogyrus cichlidarum* gen. nov., sp. nov., Urogyridae fam. nov., monogène parasite de la vessie urinaire de poissons cichlidés au Cameroun. Canadian Journal of Zoology, 72, 561–566.

[3] Bilong Bilong CF, Euzet L. 1995. *Onchobdella bopeleti* n. sp. (Monogenea, Ancyrocephalidae) parasite branchial de *Hemichromis fasciatus* (Peters, 1857) (Cichlidae). Journal of African Zoology, 109, 253–258.

[4] Bitja-Nyom AR, Agnèse JF, Pariselle A, Bilong Bilong CF, Gilles A, Snoeks J. 2021. A systematic revision of the five-spotted *Hemichromis* complex (Cichliformes: Cichlidae) from West Africa and Lower Guinea, with the description of a new species from Cameroon. Hydrobiologia, 5, 3779–3803.

[5] Bitjanyom AR, Gilles A, Pariselle A, Snoeks J, Bilong Bilong CF. 2012. Divergences morphologiques allopatriques et sympatriques de *Tilapia kottae* (Perciformes, Cichlidae) endémique des lacs Barombi Kotto et Mboandong et affinités avec des tilapias de la ligne volcanique du Cameroun. Cybium, 36, 335–348.

[6] Braga MP, Razzolini E, Boeger WA. 2015. Drivers of parasite sharing among neotropical freshwater fishes. Journal of Animal Ecology, 84, 487–497. 25283218 10.1111/1365-2656.12298

[7] Brooks D, McLennan D. 1993. Parascript. Parasites and the language of evolution. Smithsonian Institution Press: Washington, DC, USA.

[8] Brooks DR, Hoberg EP, Boeger WA. 2019. The Stockholm Paradigm: climate change and emerging disease. University of Chicago Press: Chicago, IL, USA.

[9] Clayton DH, Al-Tamini S, Johnson KP. 2003. The ecological basis of coevolutionary history, in Tangled trees: phylogeny, cospeciation, and coevolution. Page RDM, Editor. University of Chicago Press: Chicago, IL, USA. p. 310–341.

[10] Cruz-Laufer AJ, Artois T, Koblmüller S, Pariselle A, Smeets K, Van Steenberge M, Vanhove MPM. 2022. Explosive networking: the role of adaptive host radiations and ecological opportunity in a species-rich host-parasite assembly. Ecology Letters, 25, 1795–1812. 35726545 10.1111/ele.14059

[11] Cruz-Laufer AJ, Artois T, Smeets K, Pariselle A, Vanhove MPM. 2021. The cichlid–*Cichlidogyrus* network: a blueprint for a model system of parasite evolution. Hydrobiologia, 848, 3847–3863.

[12] Cruz-Laufer AJ, Pariselle A, Jorissen MWP, Muterezi Bukinga F, Al Assadi A, Van Steenberge M, Koblmüller S, Sturmbauer C, Smeets K, Huyse T, Artois T, Vanhove MPM. 2022. Somewhere I belong: phylogeny and morphological evolution in a species-rich lineage of ectoparasitic flatworms infecting cichlid fishes. Cladistics, 38, 465–512. 35488795 10.1111/cla.12506

[13] de Meeûs T, Michalakis Y, Renaud F. 1998. Santa Rosalia revisited: or why are there so many kinds of parasites in “The Garden of Earthly Delights”? Parasitology Today, 14, 10–13. 17040683 10.1016/s0169-4758(97)01163-0

[14] Dossou C. 1982. Parasites de poissons d’eau douce du Bénin III. Espèces nouvelles du genre *Cichlidogyrus* (Monogenea) parasites de Cichlidae. Bulletin de l’Institut Fondamental d’Afrique Noire, 44, 295–322.

[15] Dossou S. 1973. Recherches sur les monogènes parasites des poissons d’eau douce du Sud Dahomey. Doctoral dissertation. Université des Sciences et Techniques du Languedoc and Université du Dahomey.

[16] Dunz AR, Schliewen UK. 2013. Molecular phylogeny and revised classification of the haplotilapiine cichlid fishes formerly referred to as “Tilapia”. Molecular Phylogenetics and Evolution, 68, 64–80. 23542002 10.1016/j.ympev.2013.03.015

[17] Fankoua S-O, Bitja Nyom AR, Bahanak DND, Bilong Bilong CF, Pariselle A. 2017. Influence of preservative and mounting media on the size and shape of monogenean sclerites. Parasitology Research, 116, 2277–2281. 28667521 10.1007/s00436-017-5534-7

[18] Franceschini L, Zago AC, Müller MI, Francisco CJ, Takemoto RM, da Silva RJ. 2018. Morphology and molecular characterization of *Demidospermus spirophallus* n. sp., *D. prolixus* n. sp. (Monogenea: Dactylogyridae) and a redescription of *D. anus* in siluriform catfish from Brazil. Journal of Helminthology, 92, 228–243. 28382887 10.1017/S0022149X17000256

[19] Froese R, Pauly D. 2000. FishBase 2000: concepts, designs and data sources. ICLARM: Los Baños, Laguna, Philippines.

[20] Gandon S, Michalakis Y. 2002. Local adaptation, evolutionary potential and host-parasite coevolution: interactions between migration, mutation, population size and generation time. Journal of Evolutionary Biology, 15, 451–462.

[21] Geraerts M, Huyse T, Barson M, Bassirou H, Bilong Bilong CF, Bitja Nyom AR, Chocha Manda A, Cruz-Laufer AJ, Kalombo Kabalika C, Kapepula Kasembele G, Muterezi Bukinga F, Njom S, Artois T, Vanhove MPM. 2022. Mosaic or melting pot: the use of monogeneans as a biological tag and magnifying glass to discriminate introduced populations of Nile tilapia in sub-Saharan Africa. Genomics, 114, 110328. 35276332 10.1016/j.ygeno.2022.110328

[22] Gibson DI, Timofeeva TA, Gerasev PI. 1996. A catalogue of the nominal species of the monogenean genus *Dactylogyrus* Diesing, 1850 and their host genera. Systematic Parasitology, 35, 3–48.

[23] Giraud T. 2006. Speciation in parasites: host switching does not automatically lead to allopatry. Trends in Parasitology, 22, 151–152. 16488190 10.1016/j.pt.2006.02.001

[24] Gittenberger A, Gittenberger E. 2011. Cryptic, adaptive radiation of endoparasitic snails: sibling species of *Leptoconchus* (Gastropoda: Coralliophilidae) in corals. Organisms Diversity and Evolution, 11, 21–41.

[25] Gobbin TP, Vanhove MPM, Seehausen O, Maan ME. 2021. Microhabitat distributions and species interactions of ectoparasites on the gills of cichlid fish in Lake Victoria, Tanzania. International Journal for Parasitology, 51, 201–214. 33161003 10.1016/j.ijpara.2020.09.001

[26] Gobbin TP, Vanhove MPM, Seehausen O, Maan ME, Pariselle A. 2021. Four new species of *Cichlidogyrus* (Platyhelminthes, Monogenea, Dactylogyridae) from Lake Victoria haplochromine cichlid fishes, with the redescription of *C. bifurcatus* and *C. longipenis*. bioRxiv. 10.1101/2021.01.29.428376. PMC11305117 39109983

[27] Goloboff PA. 2014. Extended implied weighting. Cladistics, 30, 260–272. 34788977 10.1111/cla.12047

[28] Goloboff PA. 1993. Estimating character weights during tree search. Cladistics, 9, 83–91. 34929936 10.1111/j.1096-0031.1993.tb00209.x

[29] Goloboff PA. 1999. Analyzing large data sets in reasonable times: solutions for composite optima. Cladistics, 15, 415–428. 34902941 10.1111/j.1096-0031.1999.tb00278.x

[30] Goloboff PA. 2003. Improvements to resampling measures of group support. Cladistics, 19, 324–332.

[31] Goloboff PA, Carpenter JM, Arias JS, Esquivel DRM. 2008. Weighting against homoplasy improves phylogenetic analysis of morphological data sets. Cladistics, 24, 758–773.

[32] Goloboff PA, Catalano SA. 2016. TNT version 1.5, including a full implementation of phylogenetic morphometrics. Cladistics, 32, 221–238. 34727670 10.1111/cla.12160

[33] Goloboff PA, Mattoni CI, Quinteros AS. 2006. Continuous characters analyzed as such. Cladistics, 22, 589–601. 34892898 10.1111/j.1096-0031.2006.00122.x

[34] Guégan J-F, Agnèse J-F. 1991. Parasite evolutionary events inferred from host phylogeny: the case of *Labeo* species (Teleostei, Cyprinidae) and their dactylogyrid parasites (Monogenea, Dactylogyridae). Canadian Journal of Zoology, 69, 595–603.

[35] Hahn C, Weiss SJ, Stojanovski S, Bachmann L. 2015. Co-speciation of the ectoparasite *Gyrodactylus teuchis* (Monogenea, Platyhelminthes) and its salmonid hosts. PLoS One, 10, e0127340. 26080029 10.1371/journal.pone.0127340 PMC4469311

[36] Humason G. 1979. Animal tissue techniques, 4th edn. Freeman and Company: San Francisco, CA, USA.

[37] Huyse T, Poulin R, Théron A. 2005. Speciation in parasites: a population genetics approach. Trends in Parasitology, 21, 469–475. 16112615 10.1016/j.pt.2005.08.009

[38] ICZN. 1999. International Code of Zoological Nomenclature, 4th edn. London, United Kingdom: International Trust for Zoological Nomenclature.

[39] Jorge F, Poulin R. 2018. Poor geographical match between the distributions of host diversity and parasite discovery effort. Proceedings of the Royal Society B: Biological Sciences, 285, 20180072. 10.1098/rspb.2018.0072 PMC5998099 29848643

[40] Jorissen M, Vanhove MPM, Pariselle A, Snoeks J, Vreven E, Šimková A, Wamuini Lunkayilakio S, Manda AC, Kapepula Kasembele G, Muterezi Bukinga F, Artois T, Huyse T. 2022. Molecular footprint of parasite co-introduction with Nile tilapia in the Congo Basin. Organisms Diversity & Evolution, 22, 1003–1019.

[41] Jorissen MWP, Huyse T, Pariselle A, Wamuini Lunkayilakio S, Muterezi Bukinga F, Chocha Manda A, Kapepula Kasembele G, Vreven EJ, Snoeks J, Decru E, Artois T, Vanhove MPM. 2020. Historical museum collections help detect parasite species jumps after tilapia introductions in the Congo Basin. Biological Invasions, 11, 1123.

[42] Jorissen MWP, Pariselle A, Huyse T, Vreven EJ, Snoeks J, Volckaert FAM, Chocha Manda A, Kasembele GK, Artois T, Vanhove MPM. 2018. Diversity and host specificity of monogenean gill parasites (Platyhelminthes) of cichlid fishes in the Bangweulu-Mweru ecoregion. Journal of Helminthology, 92, 417–437. 28829000 10.1017/S0022149X17000712

[43] Jorissen MWP, Pariselle A, Huyse T, Vreven EJ, Snoeks J, Decru E, Kusters T, Lunkayilakio SW, Bukinga FM, Artois T, Vanhove MPM. 2018. Six new dactylogyrid species (Platyhelminthes, Monogenea) from the gills of cichlids (Teleostei, Cichliformes) from the Lower Congo Basin. Parasite, 25, 64. 30526819 10.1051/parasite/2018059 PMC6284406

[44] Kadlec D, Šimková A, Gelnar M. 2003. The microhabitat distribution of two *Dactylogyrus* species parasitizing the gills of the barbel, *Barbus barbus*. Journal of Helminthology, 77, 317–325. 14627448 10.1079/joh2003183

[45] Kawecki TJ. 1998. Red queen meets Santa Rosalia: arms race and the evolution of host specialization in organisms with parasitic lifestyles. American Naturalist, 152, 635–651. 10.1086/286195 18811369

[46] Khang TF, Soo OYM, Tan WB, Lim LHS. 2016. Monogenean anchor morphometry: systematic value, phylogenetic signal, and evolution. PeerJ, 4, e1668. 26966649 10.7717/peerj.1668 PMC4783769

[47] Klassen GJ, Beverley-Burton M. 1987. Phylogenetic relationships of *Ligictaluridus* spp. (Monogenea: Ancyrocephalidae) and their ictalurid (Siluriformes) hosts: an hypothesis. Proceedings of the Helminthological Society of Washington, 54, 84–90.

[48] Klassen GJ. 1994. Phylogeny of *Haliotrema* species (Monogenea: Ancyrocephalidae) from boxfishes (Tetraodontiformes: Ostraciidae): Are *Haliotrema* species from boxfishes monophyletic? Journal of Parasitology, 80, 596–610. 8064528

[49] Kmentová N, Cruz-Laufer AJ, Pariselle A, Smeets K, Artois T, Vanhove MPM. 2022. Dactylogyridae 2022: a meta-analysis of phylogenetic studies and generic diagnoses of parasitic flatworms using published genetic and morphological data. International Journal for Parasitology, 52, 427–457. 35245493 10.1016/j.ijpara.2022.01.003

[50] Kmentová N, Koblmüller S, Van Steenberge M, Raeymaekers JAM, Artois T, De Keyzer ELR, Milec L, Muterezi Bukinga F, Mulimbwa N’sibula T, Masilya Mulungula P, Ntakimazi G, Volckaert FAM, Gelnar M, Vanhove MPM. 2020. Weak population structure and recent demographic expansion of the monogenean parasite *Kapentagyrus* spp. infecting clupeid fishes of Lake Tanganyika, East Africa. International Journal for Parasitology, 50, 471–486. 32277985 10.1016/j.ijpara.2020.02.002

[51] Kuhn T, García-Màrquez J, Klimpel S. 2011. Adaptive radiation within marine anisakid nematodes: a zoogeographical modeling of cosmopolitan, zoonotic parasites. PLoS One, 6, e28642. 22180787 10.1371/journal.pone.0028642 PMC3236750

[52] Lagrue C, Poulin R. 2016. The scaling of parasite biomass with host biomass in lake ecosystems: Are parasites limited by host resources? Ecography, 39, 507–514.

[53] Lamboj A. 2009. A new dwarf cichlid genus and species (Teleostei, Cichlidae) from Guinea, West Africa. Zootaxa, 2173, 41–48.

[54] Lamboj A. 2001. Revision des *Chromidotilapia batesii/finleyi*-Komplexes (Teleostei, Perciformes), mit der Beschreibung einer neuen Gattung und dreier neuer Arten. Verhandlungen der Gesellschaft für Ichthyologie, 2, 11–47.

[55] Lamboj A. 2004. The cichlid fishes of Western Africa. Birgit Schmettkamp Verlag: Bornheim, Germany.

[56] Lamboj A, Bartel D, Emiliano Dell’ampio E. 2014. Revision of the *Pelvicachromis taeniatus*-group (Perciformes), with revalidation of the taxon *Pelvicachromis kribensis* (Boulenger, 1911) and the description of a new species. Cybium, 38, 205–222.

[57] Landis JR, Koch GG. 1977. The measurement of observer agreement for categorical data. Biometrics, 33, 159. 843571

[58] Liaw A, Wiener M. 2002. Classification and regression by randomForest. R News, 2, 18–22.

[59] Lim GS, Balke M, Meier R. 2012. Determining species boundaries in a world full of rarity: singletons, species delimitation methods. Systematic Biology, 61, 165–169. 21482553 10.1093/sysbio/syr030

[60] Llewellyn L. 1963. Larvae and larval development of monogeneans. Advances in Parasitology, 1, 287–326. 14117622 10.1016/s0065-308x(08)60506-0

[61] Lumme J, Ziętara MS, Lebedeva D. 2017. Ancient and modern genome shuffling: reticulate mito-nuclear phylogeny of four related allopatric species of *Gyrodactylus* von Nordmann, 1832 (Monogenea: Gyrodactylidae), ectoparasites on the Eurasian minnow *Phoxinus phoxinus* (L.) (Cyprinidae). Systematic Parasitology, 94, 183–200. 28130668 10.1007/s11230-016-9696-y

[62] Martin CH, Cutler JS, Friel JP, Dening Touokong C, Coop G, Wainwright PC. 2015. Complex histories of repeated gene flow in Cameroon crater lake cichlids cast doubt on one of the clearest examples of sympatric speciation. Evolution, 69, 1406–1422. 25929355 10.1111/evo.12674

[63] Matejusová I, Simková A, Sasal P, Gelnar M. 2003. Microhabitat distribution of *Pseudodactylogyrus anguillae* and *Pseudodactylogyrus bini* among and within gill arches of the European eel (*Anguilla anguilla* L.). Parasitology Research, 89, 290–296. 12632166 10.1007/s00436-002-0682-8

[64] McCoy KD. 2003. Sympatric speciation in parasites – What is sympatry? Trends in Parasitology, 19, 400–404. 12957516 10.1016/S1471-4922(03)00194-6 PMC7129588

[65] Mendlová M, Pariselle A, Vyskočilová M, Šimková A. 2010. Molecular phylogeny of monogeneans parasitizing African freshwater Cichlidae inferred from LSU rDNA sequences. Parasitology Research, 107, 1405–1413. 20697913 10.1007/s00436-010-2008-6

[66] Messu Mandeng FD, Bilong Bilong CF, Pariselle A, Vanhove MPM, Bitja Nyom AR, Agnèse JF. 2015. A phylogeny of *Cichlidogyrus* spp. (Monogenea, Dactylogyridea) clarifies a host-switch between fish families and reveals an adaptive component to attachment organ morphology of this parasite genus. Parasites & Vectors, 8, 582. 26554914 10.1186/s13071-015-1181-y PMC4641334

[67] Meyer D, Dimitriadou E, Hornik K, Weingessel A, Leisch F. 2020. e1071: misc functions of the Department of Statistics, Probability Theory Group (Formerly: E1071), TU Wien: R package version 1.7-4. Department of Statistics, Probability Theory Group, TU Wien: Vienna, Austria.

[68] Mirande JM. 2009. Weighted parsimony phylogeny of the family Characidae (Teleostei: Characiformes). Cladistics, 25, 574–613. 34879592 10.1111/j.1096-0031.2009.00262.x

[69] Musilova Z, Indermaur A, Bitja-Nyom AR, Omelchenko D, Kłodawska M, Albergati L, Remišová K, Salzburger W. 2019. Evolution of the visual sensory system in cichlid fishes from crater lake Barombi Mbo in Cameroon. Molecular Ecology, 28, 5010–5031. 31472098 10.1111/mec.15217

[70] Orr MR, Smith TB. 1998. Ecology and speciation. Trends in Ecology & Evolution, 13, 502–506. 21238408 10.1016/s0169-5347(98)01511-0

[71] Page RDM, Lee PLM, Becher SA, Griffiths R, Clayton DH. 1998. A different tempo of mitochondrial DNA evolution in birds and their parasitic lice. Molecular Phylogenetics and Evolution, 9, 276–293. 9562986 10.1006/mpev.1997.0458

[72] Paperna I. 1968. *Onchobdella* n. gen. New Genus of monogenetic trematodes (Dactylogyridae, Bychowski 1933) from cichlid fish from West Africa. Proceedings of the Helminthological Society of Washington, 35, 200–206.

[73] Paperna I. 1960. Studies on monogenetic trematodes in Israel. 2. Monogenetic trematodes of Cichlids. Bamidgeh, 12, 20–33.

[74] Paperna I. 1969. Monogenetic trematodes of the fish of the Volta basin and South Ghana. Bulletin de l’Institut Fondamental d’Afrique Noire, Série A, Sciences Naturelles, 31, 840–880.

[75] Paperna I. 1979. Monogenea of inland water fish in Africa. Annales du Musée Royal de l’Afrique Centrale Tervuren Belgique Serie IN-8 Science Zoologiques, 226, 1–131.

[76] Pariselle A. 1995. Études des parasites de Cichlidae en Afrique de l’Ouest, in Comptes Rendus de l’Atelier Biodiversité et Aquaculture. Abidjan 21/25 Novembre 1994, Agnèse JF, Editor. CRO: Abidjan, Côte d’Ivoire. p. 44–52.

[77] Pariselle A, Bilong Bilong CF, Euzet L. 2003. Four new species of *Cichlidogyrus* Paperna, 1960 (Monogenea, Ancyrocephalidae), all gill parasites from African mouthbreeder tilapias of the genera *Sarotherodon* and *Oreochromis* (Pisces, Cichlidae), with a redescription of *C. thurstonae* Ergens, 1981. Systematic Parasitology, 56, 201–210. 14707506 10.1023/b:sypa.0000003807.27452.bd

[78] Pariselle A, Bitja Nyom AR, Bilong Bilong CF. 2014. Four new species of *Cichlidogyrus* (Monogenea, Ancyrocephalidae) from *Sarotherodon mvogoi* and *Tylochromis sudanensis* (Teleostei, Cichlidae) in Cameroon. Zootaxa, 3881, 258–266. 25543634 10.11646/zootaxa.3881.3.4

[79] Pariselle A, Euzet L. 2009. Systematic revision of dactylogyridean parasites (Monogenea) from cichlid fishes in Africa, the Levant and Madagascar. Zoosystema, 31, 849–898.

[80] Pariselle A, Euzet L. 1996. *Cichlidogyrus* Paperna, 1960 (Monogenea, Ancyrocephalidae): gill parasites from West African Cichlidae of the subgenus *Coptodon* Regan, 1920 (Pisces), with descriptions of six new species. Systematic Parasitology, 34, 109–124.

[81] Pariselle A, Euzet L. 1997. New species of *Cichlidogyrus* Paperna, 1960 (Monogenea, Ancyrocephalidae) from the gills of *Sarotherodon occidentalis* (Daget) (Osteichthyes, Cichlidae) in Guinea and Sierra Leone (West Africa). Systematic Parasitology, 38, 221–230.

[82] Pariselle A, Euzet L. 2004. Two new species of *Cichlidogyrus* Paperna, 1960 (Monogenea, Ancyrocephalidae) gill parasites on *Hemichromis fasciatus* (Pisces, Cichlidae) in Africa, with remarks on parasite geographical distribution. parasite, 11, 359–364. 15638136 10.1051/parasite/2004114359

[83] Pariselle A, Euzet L. 1995. Trois Monogènes nouveaux parasites branchiaux de *Pelmatochromis buettikoferi* (Steindachner, 1895) (Cichlidae) en Guinée. Parasite, 2, 203–209.

[84] Pariselle A, Euzet L. 1995. *Scutogyrus* gen. n. (Monogenea: Ancyrocephalidae) for *Cichlidogyrus longicornis minus* Dossou, 1982, *C. l. longicornis*, and *C. l. gravivaginus* Paperna and Thurston, 1969, with description of three new species parasitic on African. Journal of the Helminthological Society of Washington, 62, 157–173.

[85] Pariselle A, Euzet L. 1995. Gill parasites of the genus *Cichlidogyrus* Paperna, 1960 (Monogenea, Ancyrocephalidae) from *Tilapia guineensis* (Bleeker, 1862), with descriptions of six new species. Systematic Parasitology, 30, 187–198.

[86] Pariselle A, Morand S, Deveney M, Pouyaud L. 2003. Parasite species richness of closely related hosts: historical scenario and “genetic” hypothesis, in Hommage à Louis Euzet—taxonomie, écologie et évolution des métazoaires parasites. Taxonomy, Ecology and Evolution of Metazoan Parasites. Combes C, Jourdan J, Editors. Presses Universitaires de Perpignan: Perpignan. p. 147–166.

[87] Paterson AM, Banks J. 2001. Analytical approaches to measuring cospeciation of host and parasites: through a glass, darkly. International Journal for Parasitology, 31, 1012–1022. 11406147 10.1016/s0020-7519(01)00199-0

[88] Poisot T, Desdevises Y. 2010. Putative speciation events in *Lamellodiscus* (Monogenea: Diplectanidae) assessed by a morphometric approach. Biological Journal of the Linnean Society, 99, 559–569.

[89] Poulin R. 2014. Parasite biodiversity revisited: frontiers and constraints. International Journal for Parasitology, 44, 581–589. 24607559 10.1016/j.ijpara.2014.02.003

[90] Poulin R. 2002. The evolution of monogenean diversity. International Journal for Parasitology, 32, 245–254. 11835968 10.1016/s0020-7519(01)00329-0

[91] Poulin R, Presswell B, Jorge F. 2020. The state of fish parasite discovery and taxonomy: a critical assessment and a look forward. International Journal for Parasitology, 50, 733–742. 32151615 10.1016/j.ijpara.2019.12.009

[92] Pouyaud L, Desmarais E, Deveney M, Pariselle A. 2006. Phylogenetic relationships among monogenean gill parasites (Dactylogyridea, Ancyrocephalidae) infesting tilapiine hosts (Cichlidae): systematic and evolutionary implications. Molecular Phylogenetics and Evolution, 38, 241–249. 16214376 10.1016/j.ympev.2005.08.013

[93] Price CE, Peebles HE, Bamford T. 1969. The monogenean parasites of African fishes. IV. Two new species from South African hosts. Revue de Zoologie et de Botanique Africaines, 79, 117–124.

[94] Price P. 1980. Evolutionary biology of parasites. Princeton University Press: Princeton, NJ, USA.

[95] Pugachev ON, Gerasev PI, Gussev AV, Ergens R, Khotenowsky I. 2009. Guide to the Monogenoidea of freshwater fish of the Palaearctic and Amur regions. Ledizioni-Ledi Publishing: Milano, Italy.

[96] Rahmouni C, Van Steenberge M, Vanhove MPM, Šimková A. 2021. Intraspecific morphological variation in *Cichlidogyrus* (Monogenea) parasitizing two cichlid hosts from Lake Tanganyika exhibiting different dispersal capacities. Hydrobiologia, 848, 3833–3845.

[97] Rahmouni C, Vanhove MPM, Koblmüller S, Šimková A. 2022. Molecular phylogeny and speciation patterns in host-specific monogeneans (*Cichlidogyrus*, Dactylogyridae) parasitizing cichlid fishes (Cichliformes, Cichlidae) in Lake Tanganyika. International Journal for Parasitology, 52, 359–375. 35288119 10.1016/j.ijpara.2021.12.004

[98] Rahmouni C, Vanhove MPM, Šimková A. 2018. Seven new species of *Cichlidogyrus* Paperna, 1960 (Monogenea: Dactylogyridae) parasitizing the gills of Congolese cichlids from Northern Lake Tanganyika. PeerJ, 6, e5604. 30370182 10.7717/peerj.5604 PMC6202960

[99] Rahmouni C, Vanhove MPM, Šimková A. 2017. Underexplored diversity of gill monogeneans in cichlids from Lake Tanganyika: eight new species of *Cichlidogyrus* Paperna, 1960 (Monogenea: Dactylogyridae) from the northern basin of the lake, with remarks on the vagina and the heel of the male copulatory. Parasites & Vectors, 10, 591. 29197419 10.1186/s13071-017-2460-6 PMC5712084

[100] Řehulková E, Mendlová M, Šimková A. 2013. Two new species of *Cichlidogyrus* (Monogenea: Dactylogyridae) parasitizing the gills of African cichlid fishes (Perciformes) from Senegal: morphometric and molecular characterization. Parasitology Research, 112, 1399–1410. 23403992 10.1007/s00436-013-3291-9

[101] Richards EJ, Poelstra JW, Martin CH. 2018. Don’t throw out the sympatric speciation with the crater lake water: fine-scale investigation of introgression provides equivocal support for causal role of secondary gene flow in one of the clearest examples of sympatric speciation. Evolution Letters, 2, 524–540. 30283699 10.1002/evl3.78 PMC6145409

[102] Rindoria NM, Mungai LK, Yasindi AW, Otachi EO. 2016. Gill monogeneans of *Oreochromis niloticus* (Linnaeus, 1758) and *Oreochromis leucostictus* (Trewavas, 1933) in Lake Naivasha, Kenya. Parasitology Research, 115, 1501–1508. 26691859 10.1007/s00436-015-4883-3

[103] Rodríguez-González A, May-Tec AL, Herrera-Silveira J, Puch-Hau C, Quintanilla-Mena M, Villafuerte J, Velázquez-Abunader I, Aguirre-Macedo ML, Vidal-Martínez VM. 2020. Fluctuating asymmetry of sclerotized structures of *Haliotrematoides* spp. (Monogenea: Dactylogyridae) as bioindicators of aquatic contamination. Ecological Indicators, 117, 106548.

[104] Rodríguez-González A, Míguez-Lozano R, Llopis-Belenguer C, Balbuena JA. 2015. Phenotypic plasticity in haptoral structures of *Ligophorus cephali* (Monogenea: Dactylogyridae) on the flathead mullet (*Mugil cephalus*): a geometric morphometric approach. International Journal for Parasitology, 45, 295–303. 25736600 10.1016/j.ijpara.2015.01.005

[105] Rodríguez-González A, Sarabeev V, Balbuena JA. 2017. Evolutionary morphology in shape and size of haptoral anchors in 14 *Ligophorus* spp. (Monogenea: Dactylogyridae). PLoS One, 12, e0178367. 28542570 10.1371/journal.pone.0178367 PMC5443544

[106] Rohde K. 1977. Noncompetitive mechanisms responsible for restricting niches. Zoologischer Anzeiger, 199, 164–172.

[107] Rohde K. 1979. A critical evaluation of intrinsic and extrinsic factors responsible for niche restriction in parasites. American Naturalist, 114, 648–671.

[108] Ronco F, Büscher HH, Indermaur A, Salzburger W. 2020. The taxonomic diversity of the cichlid fish fauna of ancient Lake Tanganyika, East Africa. Journal of Great Lakes Research, 46, 1067–1078. 33100489 10.1016/j.jglr.2019.05.009 PMC7574848

[109] Ronco F, Matschiner M, Böhne A, Boila A, Büscher HH, El Taher A, Indermaur A, Malinsky M, Ricci V, Kahmen A, Jentoft S, Salzburger W. 2021. Drivers and dynamics of a massive adaptive radiation in cichlid fishes. Nature, 589, 76–81. 33208944 10.1038/s41586-020-2930-4

[110] Schedel FDB, Musilova Z, Schliewen UK. 2019. East African cichlid lineages (Teleostei: Cichlidae) might be older than their ancient host lakes: new divergence estimates for the East African cichlid radiation. BMC Evolutionary Biology, 19, 94. 31023223 10.1186/s12862-019-1417-0 PMC6482553

[111] Schwarzer J, Lamboj A, Langen K, Misof B, Schliewen UK. 2015. Phylogeny and age of chromidotilapiine cichlids (Teleostei: Cichlidae). Hydrobiologia, 748, 185–199.

[112] Schwarzer J, Misof B, Ifuta SN, Schliewen UK. 2011. Time and origin of cichlid colonization of the lower Congo rapids. PLoS One, 6, e22380. 21799840 10.1371/journal.pone.0022380 PMC3140524

[113] Schwarzer J, Misof B, Tautz D, Schliewen UK. 2009. The root of the East African cichlid radiations. BMC Evolutionary Biology, 9, 186. 19656365 10.1186/1471-2148-9-186 PMC2739198

[114] Seehausen O. 2006. African cichlid fish: a model system in adaptive radiation research. Proceedings of the Royal Society B: Biological Sciences, 273, 1987–1998. 10.1098/rspb.2006.3539 PMC1635482 16846905

[115] Šimková A, Desdevises Y, Gelnar M, Morand S. 2000. Co-existence of nine gill ectoparasites (*Dactylogyrus*: Monogenea) parasitising the roach (*Rutilus rutilus* L.): history and present ecology. International Journal for Parasitology, 30, 1077–1088. 10996326 10.1016/s0020-7519(00)00098-9

[116] Šimková A, Morand S, Jobet E, Gelnar M, Verneau O. 2004. Molecular phylogeny of congeneric monogenean parasites (*Dactylogyrus*): a case of intrahost speciation. Evolution, 58, 1001–1018. 15212381 10.1111/j.0014-3820.2004.tb00434.x

[117] Šimková A, Řehulková E, Rasoloariniaina JR, Jorissen MWP, Scholz T, Faltýnková A, Mašová Š, Vanhove MPM. 2019. Transmission of parasites from introduced tilapias: a new threat to endemic Malagasy ichthyofauna. Biological Invasions, 21, 803–819.

[118] Singh P, Irisarri I, Torres-Dowdall J, Thallinger GG, Svardal H, Lemmon EM, Lemmon AR, Koblmüller S, Meyer A, Sturmbauer C. 2022. Phylogenomics of trophically diverse cichlids disentangles processes driving adaptive radiation and repeated trophic transitions. Ecology and Evolution, 12, e9077. 35866021 10.1002/ece3.9077 PMC9288888

[119] Stacklies W, Redestig H, Scholz M, Walther D, Selbig J. 2007. pcaMethods – a bioconductor package providing PCA methods for incomplete data. Bioinformatics, 23, 1164–1167. 17344241 10.1093/bioinformatics/btm069

[120] Stiassny MLJ, Schliewen UK. 2007. *Congochromis*, a new cichlid genus (Teleostei: Cichlidae) from Central Africa, with the description of a new species from the upper Congo River, Democratic Republic of Congo. American Museum Novitates, 3576, 1–14.

[121] Thieme ML, Abell R, Stiassny MLJ, Skelton P, Lehner B, Teugels GG, Dinerstein E, Toham AK, Burgess N, Olson D. 2005. Freshwater ecoregions of Africa and Madagascar: A conservation assessment. Island Press: Washington, USA.

[122] Trewavas E. 1974. The freshwater fishes of Rivers Mungo and Meme and Lakes Kotto, Mboandong and Soden, West Cameroon. Bulletin of the British Museum of Natural History Zoology, 26, 329–419.

[123] Ude GN, Igwe DO, Brown C, Jackson M, Bangura A, Ozokonkwo-Alor O, Ihearahu OC, Chosen O, Okoro M, Ene C, Chieze V, Unachukwu M, Onyia C, Acquaah G, Ogbonna J, Das A. 2020. DNA barcoding for identification of fish species from freshwater in Enugu and Anambra States of Nigeria. Conservation Genetics Resources, 12, 643–658.

[124] Van Steenberge M, Pariselle A, Huyse T, Volckaert FAM, Snoeks J, Vanhove MPM. 2015. Morphology, molecules, and monogenean parasites: an example of an integrative approach to cichlid biodiversity. PLoS One, 10, e0124474. 25923665 10.1371/journal.pone.0124474 PMC4414595

[125] Vanhove MPM, Hablützel PI, Pariselle A, Šimková A, Huyse T, Raeymaekers JAM. 2016. Cichlids: a host of opportunities for evolutionary parasitology. Trends in Parasitology, 32, 820–832. 27595383 10.1016/j.pt.2016.07.002

[126] Venables WN, Ripley BD. 2002. Modern applied statistics with S, 4th edn. New York, NY, USA: Springer.

[127] Vignon M, Pariselle A, Vanhove MPM. 2011. Modularity in attachment organs of African *Cichlidogyrus* (Platyhelminthes: Monogenea: Ancyrocephalidae) reflects phylogeny rather than host specificity or geographic distribution. Biological Journal of the Linnean Society, 102, 694–706.

[128] Walsh G, Lamboj A, Stiassny MLJ. 2020. Review of the cichlid genus *Thysochromis* (Teleostei: Ovalentaria) with the description of a new species from the Kouilou Province in the Republic of Congo, west–Central Africa. Journal of Fish Biology, 96, 1176–1185. 31583705 10.1111/jfb.14144

[129] Wood CL, Vanhove MPM. 2022. Is the world wormier than it used to be? We’ll never know without natural history collections. Journal of Animal Ecology, 92, 250–262. 35959636 10.1111/1365-2656.13794

[130] Wright K. 2021. nipals: Principal Components Analysis using NIPALS or Weighted EMPCA, with Gram-Schmidt Orthogonalization. R package version 0.8. https://CRAN.R-project.org/package=nipals.

[131] Zahradníčková P, Barson M, Luus-Powell WJ, Přikrylová I. 2016. Species of *Gyrodactylus* von Nordmann, 1832 (Platyhelminthes: Monogenea) from cichlids from Zambezi and Limpopo river basins in Zimbabwe and South Africa: evidence for unexplored species richness. Systematic Parasitology, 93, 679–700. 27522367 10.1007/s11230-016-9652-x

[132] Ziętara MS, Lumme J. 2002. Speciation by host switch and adaptive radiation in a fish parasite genus *Gyrodactylus* (Monogenea, Gyrodactylidae). Evolution, 56, 2445–2458. 12583585 10.1111/j.0014-3820.2002.tb00170.x

